# A Not Obvious Correlation Between the Structure of Green Fluorescent Protein Chromophore Pocket and Hydrogen Bond Dynamics: A Choreography From *ab initio* Molecular Dynamics

**DOI:** 10.3389/fmolb.2020.569990

**Published:** 2020-10-27

**Authors:** Federico Coppola, Fulvio Perrella, Alessio Petrone, Greta Donati, Nadia Rega

**Affiliations:** ^1^Department of Chemical Sciences, University of Naples Federico II, Naples, Italy; ^2^Center for Advanced Biomaterials for Healthcare@CRIB, Naples, Italy

**Keywords:** hydrogen bond dynamics, fluorescent proteins, *ab initio* molecular dynamics, structure-function correlation, QM/MM

## Abstract

The Green Fluorescent Protein (GFP) is a widely studied chemical system both for its large amount of applications and the complexity of the excited state proton transfer responsible of the change in the protonation state of the chromophore. A detailed investigation on the structure of the chromophore environment and the influence of chromophore form (either neutral or anionic) on it is of crucial importance to understand how these factors could potentially influence the protein function. In this study, we perform a detailed computational investigation based on the analysis of *ab-initio* molecular dynamics simulations, to disentangle the main structural quantities determining the fine balance in the chromophore environment. We found that specific hydrogen bonds interactions directly involving the chromophore (or not), are correlated to quantities, such as the volume of the cavity in which the chromophore is embedded and that it is importantly affected by the chromophore protonation state. The cross-correlation analysis performed on some of these hydrogen bonds and the cavity volume, demonstrates a direct correlation among them and we also identified the ones specifically involved in this correlation. We also found that specific interactions among residues far in the space are correlated, demonstrating the complexity of the chromophore environment and that many structural quantities have to be taken into account to properly describe and understand the main factors tuning the active site of the protein. From an overall evaluation of the results obtained in this work, it is shown that the residues which *a priori* are perceived to be spectators play instead an important role in both influencing the chromophore environment (cavity volume) and its dynamics (cross-correlations among spatially distant residues).

## 1. Introduction

**Figure 1:**
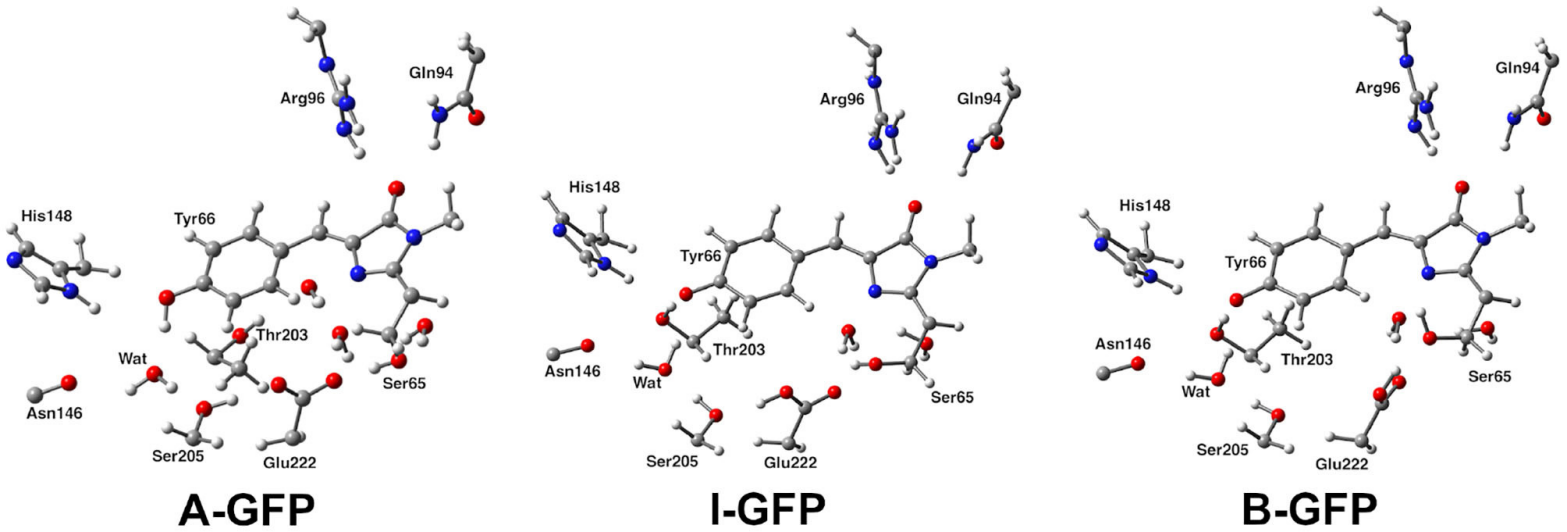
Structural comparison of GFP A, I, and B forms. The distinct protonation states between A and I/B and Glu222 reorientation between I and B can be observed.

The formation and dissociation dynamics of hydrogen-bonding is fundamental to unveil the choreography of several reactions in biology, above all, proton transfer (PT) in condensed phase (Klepeis et al., 2009; Oscar et al., 2014; Wang and Fang, 2019; Fang and Tang, 2020). PT reactions are ubiquitous in nature and are very common in photo-induced processes, such us the ones responsible of the light emission of fluorescent proteins (FPs) (Acharya et al., 2017). FPs are widely used biomarker to visualize *in vivo* processes in organisms, since their fluorescent states can be easily exploited for bioimaging techniques, as well as genetic markers in cellular biology (Chalfie et al., 1994; Tsien, 1998; Zimmer, 2002; Chen et al., 2019). The well-known green fluorescent protein (GFP) is often employed as a model system for FPs. GFP was first isolated from the jellyfish *Aequorea victoria* (Shimomura et al., 1962; Shimomura, 2005), even if many similar protein have been found in other organisms. GFP and its mutants are employed in many fields as a marker in cell biology and is responsible for an increasing interest in the whole class of fluorescent proteins that are widely investigated nowadays (Chalfie, 1995). In the wild-type GFP the chromophore 4-(p-hydroxybenzylidene)-imidazolid-5-one (HBDI) is present in the electronic ground state in both its neutral (A) and anionic deprotonated HBDI^−^ (B) form, with a neutral form population six times higher than the anionic one under physiological conditions (Chattoraj et al., 1996). The two forms give absorption bands at ~395 and 480 nm, respectively (Brejc et al., 1997; Creemers et al., 1999). After the excitation of the neutral form, an excited state proton transfer reaction takes place leading to the formation of an anionic form, denoted as I^*^. Finally the system gives a bright fluorescence near 510 nm, with a quantum yield of ~0.8 and a lifetime of ~3 ns (Striker et al., 1999; Volkmer et al., 2000). Transformations between A and B occur through an intermediate named I, indeed. This form is very similar to the neutral one, differing from A mostly for a protonation change, whereas the change from I to B is considered a slow conformational change (Figure 1) (Grigorenko et al., 2013).

Besides characterization of their functions *in vivo*, a fundamental comprehension of collective atomic motions in correlation with intramolecular and intermolecular interactions on a wide range of timescales, from femtoseconds to seconds, is the grounds for shedding light on the protein photophysics, and more in general, function. Nuclear magnetic resonance and X-ray crystallography have been useful in solving protein structures to provide models for their functions, but a highly desirable visualization of protein motions in real time can be obtained by structural dynamics techniques that are able to provide both high spatial and temporal resolutions. Usually these goals are achieved by time-resolved infrared and Raman Spectroscopies, and we refer the readers to a recent review from Fang et al. on this subject (Fang and Tang, 2020). However, the structure/spectroscopic correlation is not easy to disentangle and in this context the computational approach can be very useful to give the molecular picture behind the different spectroscopic behavior.

Although GFP has been object of several theoretical studies, focusing mostly on either the isolated chromophore (Das et al., 2003 Martin et al., 2004; Wang and Smith, 2006; Vendrell et al., 2008b; Bravaya et al., 2012; Raucci et al., 2020) or the optical properties and related proton transfer mechanism (Lill and Helms, 2002; Toniolo et al., 2004; Zhang et al., 2005; Virshup et al., 2009; Olsen et al., 2010; Bravaya et al., 2011; Conyard et al., 2011; Filippi et al., 2012; Grigorenko et al., 2013; Petrone et al., 2013, 2016a; Armengol et al., 2015; Daday et al., 2015; Nadal-Ferret et al., 2015; Shinobu and Agmon, 2017; Donati et al., 2018b; Chang et al., 2019), the concerted dynamics of the protein and its effect on the chromophore cavity has not been analyzed with the same attention yet. Fewer papers have been focused on the equilibrium properties of ground state forms (Mandal et al., 2004; Patnaik et al., 2004), mostly trying to shown the existence of a volume conserving pathway to isomerization and proving that the friction arising from the packing of the protein about the chromophore is not the only cause responsible for the dramatic effect of the protein matrix preventing the radiationless decay.

Protein hydrogen bond networks are essential to its structure, dynamics, and function. Hydrogen bond dynamics has been indeed extensively studied not only in proteins but also in fluorophores, molecular probes and DNA by a series of powerful experimental techniques, such as optical spectroscopy (Cohen et al., 2002; Pal and Zewail, 2004; Kukura et al., 2006; Frontiera and Mathies, 2007; Groot et al., 2007; Conti Nibal and Havenith, 2014; Fanetti et al., 2014; Patrizi et al., 2014), neutron scattering (Doster and Settles, 2005; Schiro et al., 2015), NMR (Nucci et al., 2011; Lewandowski et al., 2015), and by computational MD simulations (Makarov et al., 2002; Bagchi, 2005; Li et al., 2007; Amadei et a., 2011; D'Alessando et al., 2015; Donati et al., 2018b). In this context, the specific hydrogen bonding interactions and equilibrium dynamics between the GFP protein and the chromophore probably also play a significant role, although an extensive study on this complex interplay in the equilibrium ground state is still missing to the best of our knowledge, indeed. Since the necessity for providing a deeper insight into the equilibrium hydrogen bond dynamics involving HBDI and the protein pocket, in this work we use ab-initio molecular dynamics (AIMD) simulations to mainly investigate about the fluctuations of the hydrogen bond network in the different equilibrium layouts of GFP A, I, B forms. The accurate knowledge of the structural correlation within the GFP pocket dynamics in the ground state and in different forms can be informative also for understanding far from equilibrium relaxation processes in the ultrafast regime and the corresponding spectroscopic behavior. For example, ground state I form can be thought as the fluorescence decay product of the excited I^*^ state. A dynamical picture of H-bond network immediately before absorption and after emission is thus provided by a study of fluctuations around the GFP forms. Moreover, GFP represent a model system to understand how different protonation state affects the hydrogen-bond dynamics in similar systems.

AIMD trajectories are collected according to a hybrid Quantum mechanical (QM)/molecular mechanic (MM) scheme and the energy potential ruling AIMD simulations including QM and MM regions is combined according to the hybrid N-layered integrated molecular orbital and molecular mechanics (ONIOM) extrapolation scheme; more details on the simulations are provided in the Materials and Methods section (Svensson et al., 1996; Morokuma et al., 2006; Vreven et al., 2006). Density functional theory (DFT) is employed for the *ab initio* treatment of the QM part, since it has an optimal balance between accuracy and computational cost and DFT, in its hybrid version, has been vastly used for the theoretical characterization of both vibrational and dynamical properties of molecules (Wong, 1996; Adamo et al., 2001; Barone et al., 2010; Branduardi et al., 2011; Petrone et al., 2013; Cimino et al., 2016; Lingerfelt et al., 2016; Pepin et al., 2016; Wildman et al., 2019) and the description of the electronic structure of both ground and excited electronic states in macro-molecular systems of material (Hafner et al., 2006; Beaulac et al., 2011; Guido et al., 2013; Lestrange et al., 2015; Aarons et al., 2016; Chong et al., 2016; Petrone et al., 2016b, 2018; Donati et al., 2017, 2018a; Gary et al., 2017; Stein et al., 2017; Crane et al., 2019) or biological interest (Langella et al., 2002; Improta et al., 2005; Lever et al., 2014; Savarese et al., 2014; Battista et al., 2018). An *ab-initio* treatment of the HBDI chromophore and the surrounding residues is mandatory for an accurate modeling not only of their structure, but also of the non-covalent interactions among them. However, this computationally demanding approach limits the length of the collected trajectories. In particular, we chose to extract and analyze several structural parameters related to the HBDI itself and the dynamic hydrogen bond network involving its neighboring residues. Distributions are provided to show equilibrium main features of the spatial extended hydrogen bond network. Our main goal has been also to retain the temporal information provided by AIMD by performing a cross-correlation analysis to highlight the entangled and complex hydrogen bond dynamics between regions at same, and even opposite, sides with respect to the GFP chromophore, as discussed in the Results and Discussion section. As main goal, we found correlations between the hydrogen bonds dynamics and features, such as the pocket volume where it is known that this one is correlated to spectroscopic parameters involving collective low frequency motions. Conclusions and perspectives are given in the Conclusions section.

## 2. Materials and Methods

The side chain of residue Thr65 in 1EMA has been modified to Ser65 for modeling both the I and B form according to the wild type GFP. Coordinates were augmented with hydrogen atoms by employing MolProbity (Chen et al., 2010) for both the neutral and anionic forms. Arginine and Lysine residues were considered positively charged while Aspartic and Glutammic acids negatively charged according to a pH condition of about 7. We protonated six histidines in both δ and ϵ positions, while of the remaining three, two were protonated in δ (one of these is His148) and the other one in the ϵ position. As already pointed out in literature (Scharnagl et al., 1999; Oltrogge et al., 2014), His148 δ protonation is required for the interaction with HBDI Tyrosine ring. All the internal crystallographic water molecules were protonated and retained.

The protein environment was modeled recurring to the N-layered integrated molecular orbital and molecular mechanics (ONIOM) scheme, (Svensson et al., 1996; Dapprich et al., 1999; Vreven and Morokuma, 2006; Vreven et al., 2006; Clemente et al., 2010) while implicit solvent effects were taken into account by employing a polarizable continuum model partition scheme (Miertus et al., 1981; Barone and Cossi, 1998; Cossi et al., 2003; Rega et al., 2006). For all the simulations in aqueous solution, solvent was treated more specifically by the so-called ONIOM/CPCM-X (Vreven et al., 2001; Mo et al., 2004) scheme, where a solvent reaction field is computed separately in each ONIOM sub-calculation on the PCM cavity of the real system. In particular, the protein solvent accessible surface was adopted to define the PCM cavity.

**Figure 2:**
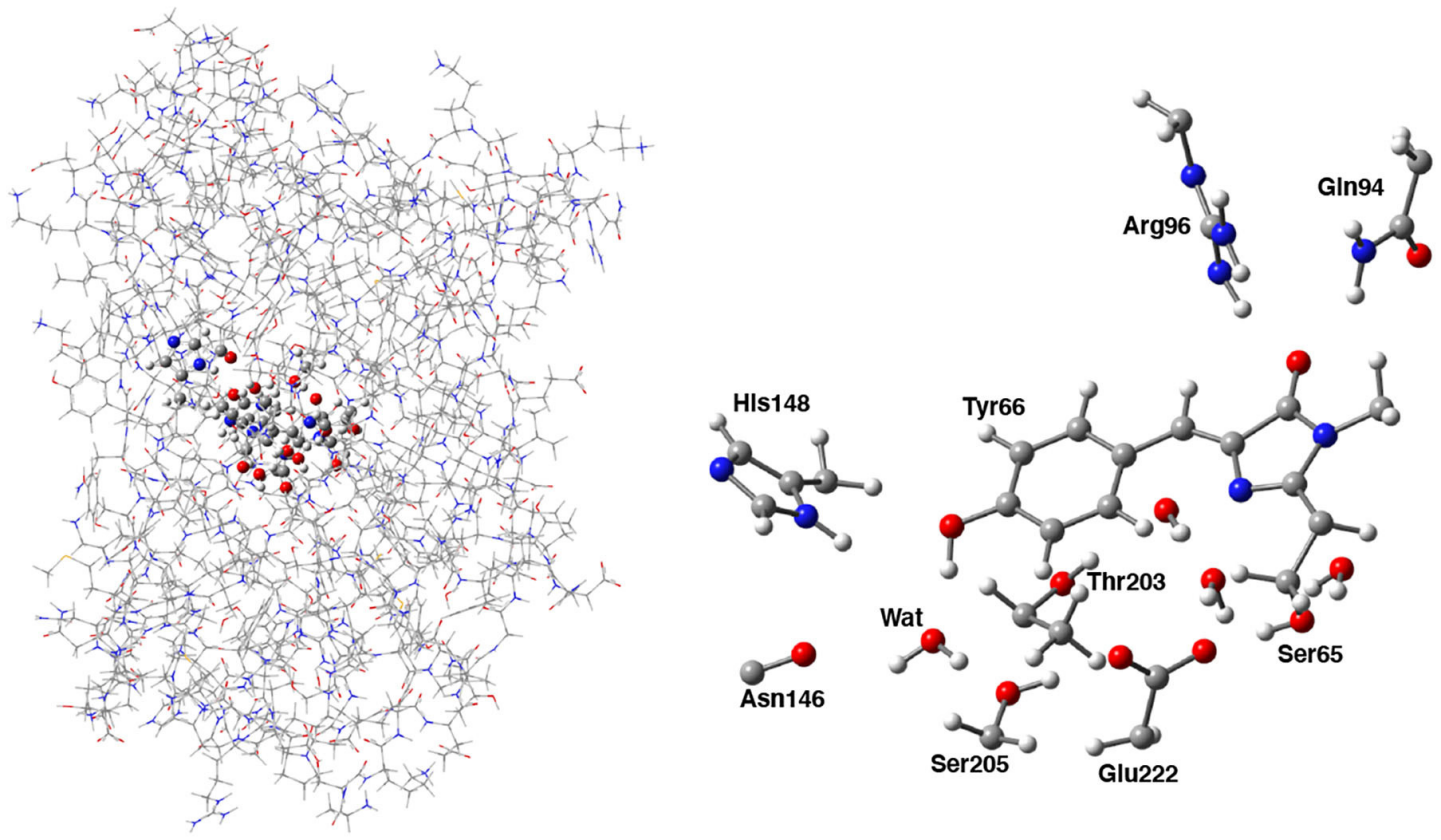
**(Left)** Hybrid ONIOM (QM/MM) partition scheme of the neutral GFP structure (PDB code:1GFL). **(Right)** ONIOM high level model (ball and stick representation) which includes the GFP chromophore, side chains of aminoacids involved in the hydrogen bond network—crystallographic water molecule, Ser205, Glu222, and Ser65—and polar fragments of residues that mimic environmental effects.

Following the ONIOM extrapolative scheme, the GFP has been then portioned into two layers, where a model region was treated at DFT theory level, while the rest of the protein, referred to as real, was represented at molecular mechanics level by employing the AMBER force field (Cornell et al., 1995). Due to the subtractive nature of the ONIOM QM/MM approach: $E^{\text{ONIOM}}=E_{\text{real}}^{\text{MM}}-E_{\text{model}}^{\text{MM}}+E_{\text{model}}^{\text{QM}}$, all the bonded (i.e., bending, stretching, torsions) force field terms are actually not required for the model (QM) part. Moreover, the treatment of the electrostatic interactions between QM and MM atoms was performed at a quantum mechanical level, introducing the charges of the MM layer into the QM model Hamiltonian (the so called “electronic embedding” scheme, as shown in Supplementary Figure 3) (Vreven et al., 2003, 2006). Non-bonding parameters are therefore the only MM parameters required for the HBDI chromophore. Following the approach of Reuter, AMBER Tyr side chain atom types were assigned to the phenol moiety of HBDI and AMBER His side chain atom types to the Imidazol HBDI ring (Reuter et al., 2002). More in details, the wt-GFP chromophore 4-(p-hydroxybenzylidene) imidazolidin-5-one (HBDI), Ser205, Glu222, Ser65, His148, Asn146, Thr203, Arg96, Gln94, and the crystallographic water molecules within a radius of 5 Å of a sphere centered on the methine bridge of the chromophore have been considered as the model region and were treated by using the range-separated version of the hybrid Becke three-parameter Lee-Yang-Parr (B3LYP) density functional (Lee et al., 1988; Becke, 1993; Stephens et al., 1994) with 6-31G(d,p) basis set (as shown in Figure 2). The rest of the protein has been modeled at molecular mechanics level. This partition scheme has been previously shown to be accurate for both ground and excited states properties (Brejc et al., 1997; Scharnagl et al., 1999; Vendrell et al., 2004, 2008a; Virshup et al., 2009; Di Donato et al., 2011; Roy et al., 2011; Wanko et al., 2012; Beerepoot et al., 2013; Petrone et al., 2013; Nadal-Ferret et al., 2015). In order to preserve the chemical nature of peptide bonds for the residues belonging to the DFT/molecular mechanics interface, the cuts have been chosen along single bonds and in general at non-polar or slightly polar bonds, preferably C–C ones. The dangling valence bonds have been capped by link hydrogen atoms.

This energy potential was used to calculate minimum energy structures in the ground state for the three forms, considered as the reference, and to run AIMD simulations within the Born-Oppenheimer approximation in the ground electronic state. More in detail, a first optimization run was performed in aqueous solution by employing the ONIOM B3LYP/6-31G(d,p)/Amber/CPCM-X level of theory followed by a further optimization run in gas phase. After this optimization procedure, the the root mean square (RMS) of the forces and displacements are within to 0.003 Ha/bohr and 0.03 bohr, respectively. In this work, since we aimed at pointing out the subtle structural differences between the three A/I/B GFP forms, from a dynamical viewpoint, ab-initio molecular dynamics simulations were performed starting from these configurations. The atom-centered density matrix propagation (ADMP) scheme was employed for this aim to allow longer simulations, since this method belonging to the extended Lagrangian approaches can exploit Gaussian basis functions and the direct propagation in time of the density matrix (Iyengar et al., 2001, 2002; Schlegel et al., 2001, 2002; Schlegel, 2003). All *ab-initio* molecular dynamics trajectories were collected using the previously validated ONIOM B3LYP/6-31G(d,p)/Amber theory level (Rega et al., 2004, 2006). Initial velocities were chosen randomly but enforcing an initial kinetic energy corresponding to the room temperature (*T* = 298 K) and individual trajectories were collected for 2.5 ps with an integration time step of 0.25 fs. This strategy allows to simultaneously sample the configurational space and dynamical room temperature fluctuations of the system, along with extracting representative correlated motions by employing the following presented analysis.

No back proton transfer event is observed during the simulation for both the I and B forms, while oscillation of the protons between the residues involved in the proton transfer is observed for the A form.

The cavity volume has been computed only for the protein pocket including the chromophore, Ser65, Ser205, and Glu222, and the crystallographic water hydrogen bonded to the phenolic oxygen of the chromophore. For each step of the trajectories for the neutral and anionic forms the cavity was computed as the resulting volume within the surface built using the overlapping spheres created by the PCM machinery on only this region [using the Universal Force Field (Rappe et al., 1992), UFF, atomic radii]. The Oxygen—Oxygen distributions between species directly involved in the proton wire have been computed with a resolution of 0.02 Å, while the cavity volume, the dihedral angles (and bond angle) have a resolution of 1 Å^3^ and 2.00°, respectively.

All the *ab initio* calculations were performed with Gaussian (Frisch et al., 2016) electronic structure software package.

Cross-correlations between the hydrogen bond distances and the pocket volume and between different hydrogen bond distances were performed for the three GFP forms. These structural quantities were extracted from the ab-initio molecular dynamics trajectories, and considering *x*(*t*) and *y*(*t*) as two distinct (average-subtracted) atom distances, their normalized cross-correlation at time-lag τ was evaluated as:

(1) $$\begin{array}{l} r_{xy}\left(\tau \right)=\frac{1}{\sqrt{\langle x^{2}\left(t\right)\rangle \langle y^{2}\left(t\right)\rangle }}\langle x\left(t+\tau \right)y\left(t\right)\rangle \end{array}$$

where the average was taken over the length of the trajectory:

(2) $$\begin{array}{l} \langle x\left(t+\tau \right)y\left(t\right)\rangle =\frac{1}{T}\int_{0}^{T}x\left(t+\tau \right)y\left(t\right)dt\approx \frac{1}{N}\overset{N}{\underset{i=1}{\sum }}x\left(t_{i}+\tau \right)y\left(t_{i}\right) \end{array}$$

The $1/\sqrt{\langle x^{2}\left(t\right)\rangle \langle y^{2}\left(t\right)\rangle }$ normalization factor (i.e., the inverse square root of the product of the two auto-correlations at τ = 0) allows *r*_*xy*_(τ) to assume values in the [−1, 1] interval. A high positive (negative) correlation *r*_*xy*_(τ) at time-lag τ suggests a high correlation between *y*(*t*) and *x*(*t*) (−*x*(*t*)), shifted by −τ along the time-axis. In order to disclose and analyse the most significant correlations between structural features of GFP pocket, only those with |*r*_*xy*_| > 0.45 were retained.

## 3. Results and Discussion

### 3.1. Analysis of Structural Parameter Distributions: A, I, B Forms

**Figure 3:**
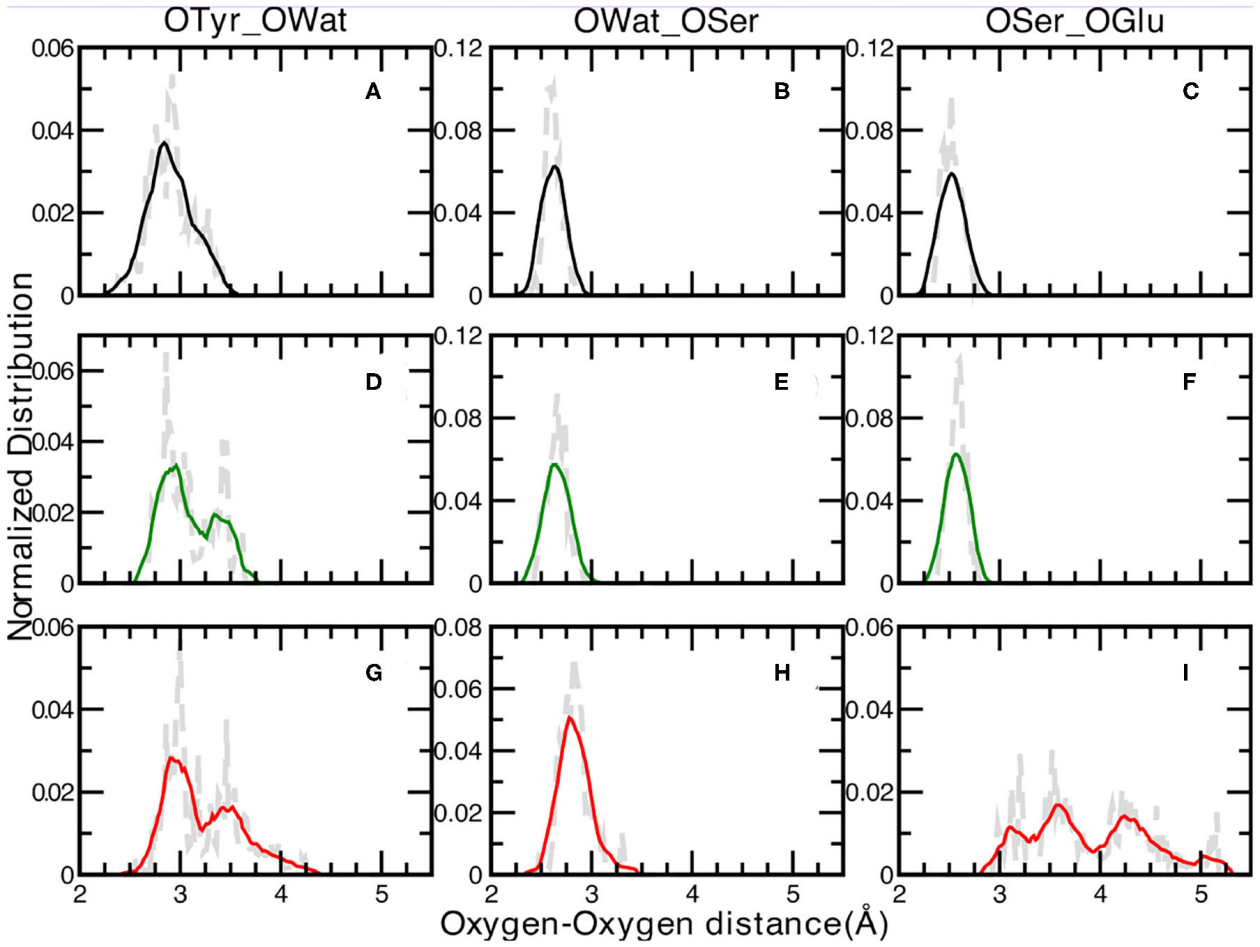
Normalized distributions of Oxygen—Oxygen distances at 0.02 Åresolution of: Tyr66—Wat [**(A,D,G)** for the A, I, and B forms, respectively], Wat—Ser205 [**(B,E,H)** for the A, I, and B forms, respectively] and Ser205—Glu222 [**(C,F,I)** for the A, I, and B forms, respectively]. Average values, shown by colored curves, are calculated every 15 points.

We report in Figure 3 the normalized distributions of oxygen—oxygen distances of the residues directly involved in the proton transfer reaction obtained from the ground state *ab-initio* molecular dynamics trajectories. The labels used in the text are shown in Figure 2. The distribution of the distances between the oxygen atoms of Tyr66 and the surrounding water molecule for the A, I and B forms are presented in Figure 3 (panels A, D, G for the A, I, and B forms, respectively). The distribution of the A form is peaked at 2.76 and 2.93 Å, covering a range of ~1Å. This result reflects small fluctuations around the average value of 2.9 Å (±0.2) calculated from the trajectory. The same distributions for the anionic I and B forms (see Figures 3D,G, respectively) show and cover an increasing range of values, from 2.65 up to 3.68 Å (Figure 3D) and from 2.57 to 4.28 Å (Figure 3G) for the I and B forms, respectively. Additionally, a more defined bimodal trend can be observed where two well-separated maxima can be recognized, a main peak at 2.86 Å (3.00 Å) and a lower one at 3.44 Å (3.46 Å) for the I (or B) form. This result proves the presence of two arrangements explored during the dynamics for both these anionic forms, less pronounced in the neutral one. By a closer inspection of the Tyr66-Wat distance dynamics in the anionic B-form (Supplementary Figure 4), it is clear that the crystallographic water molecule of the proton wire weakens its interaction with the nearby phenoxy ring of Tyr66 with respect to the other two forms. On the other hand, we observe that this water is now more involved in more stable hydrogen bonds with Asn146 and Ser205 residues. On the other side, the underlying Thr203 residue contributes to stabilize the negative charge on the chromophore.

The distribution of the distances between the oxygen atoms of water and Ser205 are analyzed next (Figures 3B,E,H for the A, I, and B forms, respectively). We observe well-defined and unimodal distributions whose maxima are centered at increasing values of 2.61, 2.66, and 2.83 Å for the three forms listed above, respectively. These distributions are also narrower with respect to the previous ones. This observation can be rationalized considering that the Ser205 residue is located on the internal region of one of the eleven β-strands that make up the GFP β-barrel, making this residue less flexible compared to Tyr66. By visually inspecting the interaction maps of the Ser205 residue with the surrounding protein residues side chains, in both neutral and anionic crystallographic forms, it is clear that the distribution around Ser205 is sharper since its environment is more characterized by hydrophobic interactions, making this residue more rigid. On the other hand, by analysing the interaction map of the Tyr66 residue, it is evident that the observed larger mobility is due by three different hydrogen bonded mutual interacting sites (His148, Thr203, and the crystallographic water molecule) which would justify the observed wider and bimodal distributions of Oxygen—Oxygen distances. The interaction maps of both the starting crystallographic structures are reported in the Supplementary Figure 11.

Moreover, the Ser205 closer environment presents no polar or charged residues that could in principle interact with its hydroxyl group, and so competing for its hydrogen bonding interactions. These two factors make Ser205 both conformationally quite rigid and stable in terms of hydrogen bond interactions. This result is also in agreement with experimental data from the crystallographic structures, indeed as in all the three simulated forms the Owat—OSer205 distances show similar values, also in the 1GFL and 1EMA (our references for the neutral and anionic forms) are very close.

A and I forms show sharp and unimodal distributions for the last OSer205—OGlu222 distance (Figures 3C,F, respectively), with two well-defined maxima centered at 2.51 Åfor the A form and at 2.60 Åfor the I form. As concerning the case of the anionic B-form (Figure 3I), this distribution shows a poorly defined shape (almost trimodal) and a wider ranges of values, starting from about 2.30 Å (already a value ~0.6 Å larger than the two previous counterparts) up to 5.18 Å. At least three regions can be recognized, centered around 3.15, 3.50, and 4.25 Å. This result supports the fact that Glu222 residue is in a less favorable orientation to interact through an hydrogen bond with the Ser205 residue in the B-form, while it is in a better arrangement to interact with the crystallographic water molecules behind the molecular plane of the HBDI chromophore (as hydrogen bond acceptor) and with Ser65 (as a donor).

Also this time we provide a closer look at Tyr66-Wat distance dynamics in the anionic B-form collected during ground state sampling as reported in Supplementary Figure 5. We can observe the environment of Tyr66 to be characterized by higher mobility of the water molecule and by the concomitant and competitive hydrogen bond interactions for this form. The negative charged chromophore in this relaxed anionic form can establish, from the Tyr66 phenoxy ring side, both a stable hydrogen bond with the hydroxy group of Thr203 and can partially interact with the NH group of His148, weakening on the contrary the interaction with nearby water molecule.

Summarizing this first analysis, the interaction between Ser205 and water are rather stable for all three forms. The chromophore water interaction shows at least two equilibrium conformations mostly for the anionic forms, while Ser205-Glu222 interactions are more stable for the A- and I-forms only, while this region starts to be more flexible in the B-form, since the Glu222 is spatially distant and more exposed to Ser65 and to two water molecules under the five-membered ring. These results clearly suggest that the chromophore in the three GFP forms is characterized by a structurally different pocket in the equilibrium simulations. The specific interactions have different equilibrium dynamics and stability in the three forms and the observed differences suggest that they have a well-defined and peculiar hydrogen bond network dynamics that will be analyzed in details in the next section.

**Figure 4:**
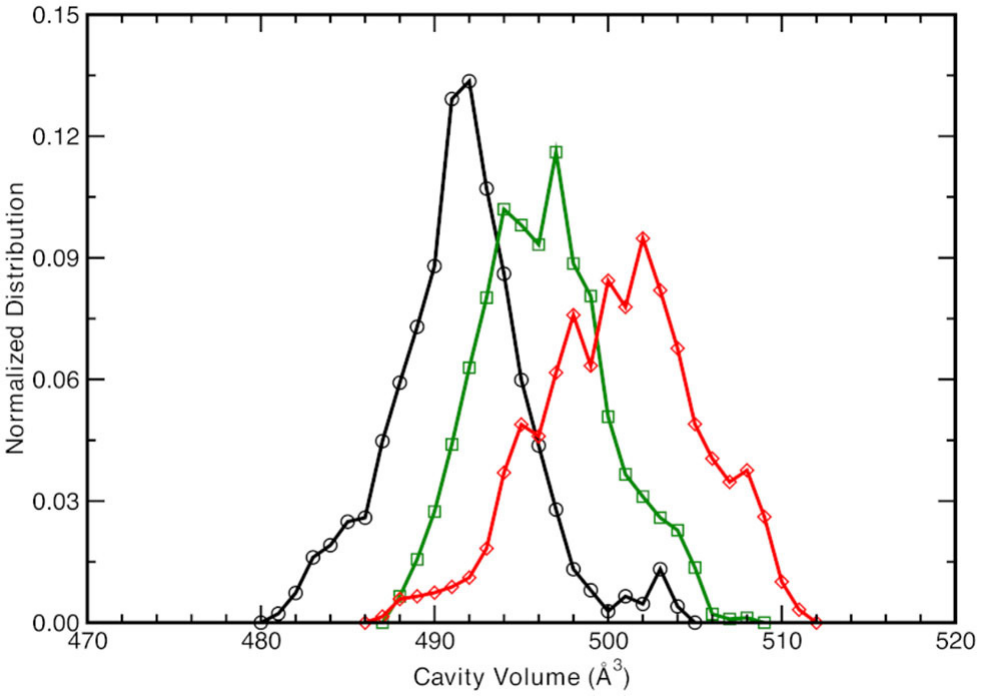
Normalized cavity volume distribution computed for each trajectory step at 1.00 Å^3^ resolution taking into account the residues participating in the hydrogen bonding network (44 atoms). Black curve refers to the neutral A-form, green and red traces refer to the anionic I- and B-form, respectively.

Given the different observed equilibrium distances of the proton wire described above, the chromophore protein pocket is investigated next. The cavity volume is computed along the trajectories according the procedure explained in the Methods section and the equilibrium distribution for the three forms is reported in Figure 4.

The neutral A form (black line) shows the sharpest distribution with a peak starting at lowest values with respect to the other two anionic forms. This suggests that the pocket is more compact in the neutral form and this observations is also supported by the previous observed narrower Oxygen—Oxygen distance distributions (see Figures 3A–C). The pocket volume distribution for the I form (dark green) is bimodal and centered at larger volume values, where there are two almost equivalent peaks (shifted of 2 and 5 Å^3^ with respect the A form). It is interesting to validate the hypothesis that these two larger values are correlated to the different hydrogen bonds arrangements discussed previously. For example, the high mobility of the crystallographic water molecule (previously observed in the OTyr66—OWat distribution of the distances in Figure 3D) can be considered to be the most important structural parameter responsible of these slightly larger pocket. The interactions for hydrogen bond network showed a weaker strength for the anionic B form in the previous analysis of distances distribution (see Figures 3G–I) and we expect a wider distribution of the volume for this last one (red curve). The protein pocket volume computed around this specific partition is wider and less sharp with multiple maxima around the main peak that is located at largest values. These trends prove that the pocket volume is directly correlated to the hydrogen bond network and dynamics, revealing how local variations in the interactions among residues are able to affect a quantity, such as the molecular volume dynamics of the protein pocket. We would also stress that from this molecular analysis based on local specific interactions, insights on larger scale quantities, such as the volume of the active site of the protein, can be obtained. On the other side, changes in volume can be interpreted and disentangled in changes on interactions localized on specific residues that are directly involved in the photo-chemistry of this system (such as the proton transfer in this case). This connection can be very useful in finding the molecular mechanisms hidden behind larger scale effects (volume changes) and can help in improving the efficiency of GFP and in general of biological molecules, when employed in fields, such as technology or drug design (Misteli and Spector, 1997; Errampalli et al., 1999; Ehrhardt, 2003; March et al., 2003; Tromans, 2004; DeLong et al., 2016).

**Figure 5:**
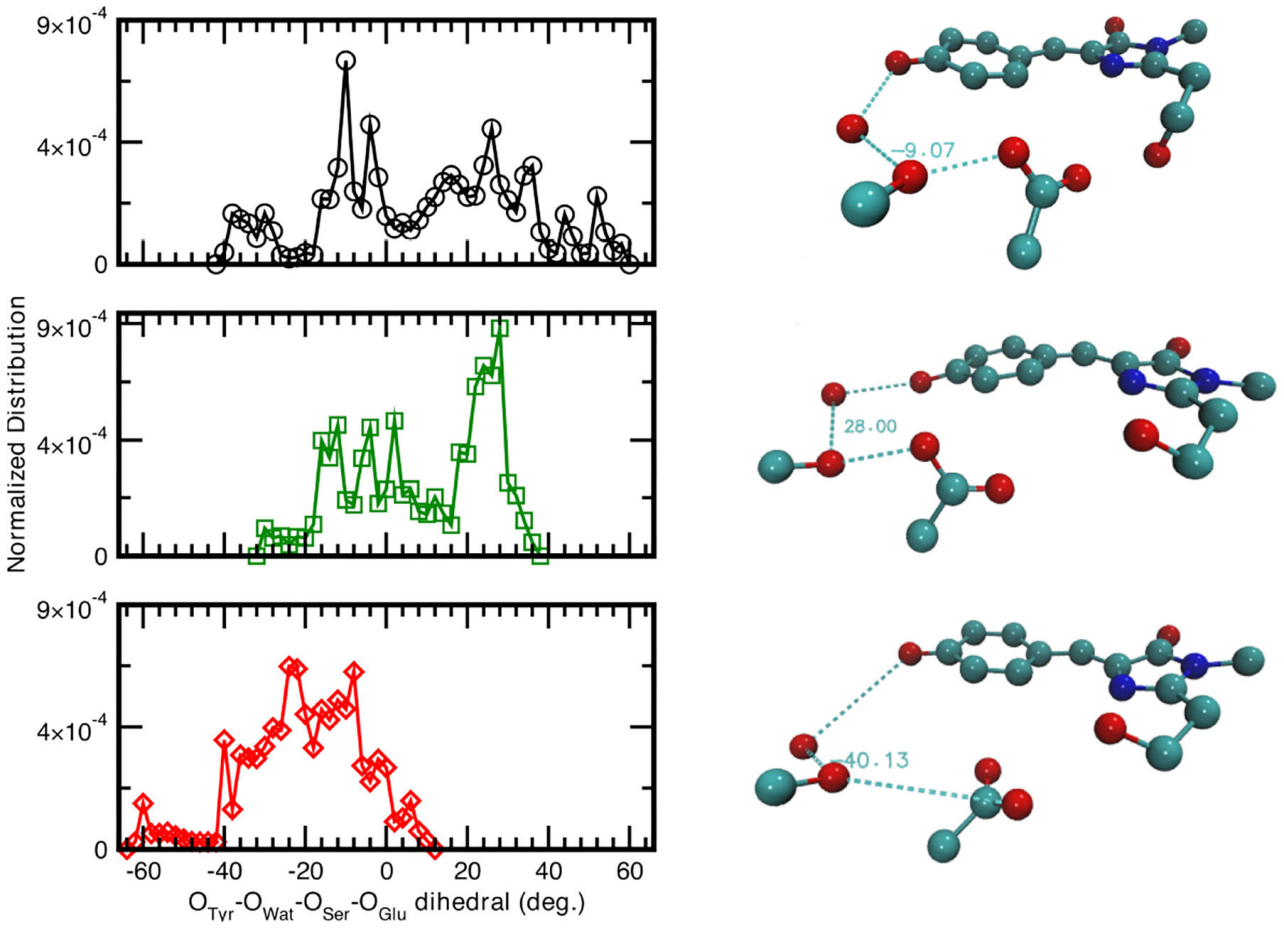
**(Left)** Normalized distribution computed from equilibrium AIMD trajectories of the dihedral angle identified by oxygens atoms belonging to the species directly involved in the H-bond chain, see X-axis label. Solid black curve and circles refer to the neutral A-form; dark green trace and squares (red trace and diamonds) refer to the anionic I-form (B-). All distributions have a resolution of 2.00°. **(Right)** Representative configuration of the neutral A-form (−9°), top panel; anionic I- (28°), middle panel and B-form (−40°), bottom panel. Numerical values are reported in degrees.

Since the distances distributions analyzed provide a piece-wise description of the hydrogen bond network involved in the PT reactivity, we also decide to report the distribution of the dihedral angle between the planes including either the oxygen atoms of the Tyr66, Wat, and Ser205 or the oxygen atoms of Wat, Ser205 and the accepting oxygen of the Glu222 in Figure 5. A wide distribution of values for the neutral A-form states that the system visits a larger conformational space ranging from −40 to 58° with a main peak at about −9° which indicated that the system oscillates already around a co-planar conformation, although in a very very flexible way. In Figure 5, we also report as example a representative configuration of the atoms in the pocket from the ground state AIMD of the neutral form (top right) in which it is possible to appreciate the relative position of the residue Glu222 with respect to the plane of the chromophore. The anionic I- and B-form dihedral distributions are different with respect to the neutral one. In the I-form, in which we observed previously that the Glu222 side-chain is more prone to a bridge interaction between the two adjacent Serine residues, the distribution is now narrower and covers a more positive range of values. This indicates that the carboxyl group of Glu222 oscillates at equilibrium from a defined co-planar network conformation (about −4°) to a limit one in which lies above the plane defined by the chromophore (28° corresponding to the maximum value of the distribution and can be seen in Figure 5, middle right). On the contrary, for the B form the hydrogen bond network dihedral distributions show mainly negative values and is 10° narrower than the previous ones (from −62 up to 10°). From a detailed analysis of the equilibrium trajectory, it has been observed that the Glu222 moves in the opposite direction from the previously discussed I-form, interacting with the water molecules below the chromophore plane (peaks at −22 and −40°), moving away from Ser205 as also schematically shown in Figure 5 (bottom right).

As also observed in the previous analyses, the distributions of the hydrogen bonds network oxygens reflect the structural rearrangement of the GFP chromophore pocket in the neutral and anionic forms. The A form is characterized by a distribution almost centered at values corresponding to a co-planar conformation, in agreement with the fact that the chromophore environment, also in the ground state, oscillates around a favorable conformation to allow the proton transfer, since this coplanarity has been shown to be important to PT reaction (Petrone et al., 2016a) In the I form case, the peak of the dihedral angles distribution is now shifted at 30°, away from a co-planar arrangement of the residues reflecting the fact that the chromophore is now anionic and the surrounding residues conformation does not need to be planar anymore. This situation is even more enhanced in the B form, where the distribution is substantially centered around non-coplanar values.

Finally, the chromophore flexibility is analyzed by inspecting the distributions of the NCCC(τ) and CCCC(ϕ) dihedral angles affecting the bridging atoms between the tyrosine and imidazolinonic ring of the HBDI chromophore in Supplementary Figure 6. On the contrary with respect to the previous dihedral, we observed here very narrow distributions centered around 0 degrees for both NCCC and CCCC cases. This supports that in the ground state HBDI chromophore is quite rigid and is not the responsible of the main observed distortion of volume of the protein pocket for the three forms. Also, the distribution of the CCC(ω) bond angle, centered at 128° for both the neutral and anionic forms, covers a narrow range of values. The stiffness of the CCC bridge states that, despite the different protonation state in the three forms, there is no mutual scissoring motions of the two chromophoric rings indeed.

To summarize, our results deriving from the study of the distributions of key structural parameters highlight the main differences of the GFP pocket in the three forms. In particular, we observe that the distributions of the distances between oxygen atoms of the proton wire residues are consistent, for all three forms, with the trend of an increasing volume of the pocket cavity. It is clear that, since the chromophore retains co-planarity in both the neutral and anionic form, the increasing trend of volumes is mainly attributable to the relative positions of the residues that participate in the proton hopping reaction.

These results underline that the photoinduced behavior of GFP is a fine balance among the residues of the pocket and of the protein matrix, being this latter the main cause preventing the photoinduced isomerization of the chromophore. Time-resolved infrared and Femtosecond stimulated Raman spectroscopy experiments, supported by theoretical works, revealed the crucial role of chromophore-Glu222 wire for the GFP fluorescence, where a concerted excited state proton transfer is hypothesized during which the protein pocket prevents the non-radiative decay (Stoner-Ma et al., 2005, 2006; Fang et al., 2009; Petrone et al., 2016a). Our finding further supports the importance the hydrogen bond network for the GFP optical properties. Previous computational and experimental works (Ormö et al., 1996; Brejc et al., 1997; Creemers et al., 1999; van Thor et al., 2002; Jung et al., 2005) have underlined the role of amino acids at position 65, 203, 222 and their impact on the optical properties of GFP (both absorption and fluorescence). Point mutations at these positions importantly affect the hydrogen bond network, determining a drastic change in the optical behavior of GFP and the excited state proton transfer reaction. As for our distributions, the role of Ser65 making an hydrogen bond with Glu222 is a crucial prerequisite for the excited state reactivity and fluorescence. Our results highlight not only the differences among the neutral and anionic forms in terms of hydrogen bonds, but also underline the difference in terms of mobility among the different hydrogen bond couples. As a main result, in agreement with experimental data, we found that it is a fine equilibrium, built of specific low energy bonds, such as the hydrogen bond, that is able to rule and change the dynamics of the GFP.

For this reasons we analyze in deep in the next section the dynamic correlation in the residues interacting with the PT hydrogen network and the chromophore.

### 3.2. Time Cross-Correlation Analysis of GFP Pocket Non-covalent Interactions

The subtle balance among non-covalent interactions in protein structures often gives rise to a complex choreography of delocalized motions (Go et al., 1983; Ichiye and Karplus, 1991; Garcia, 1992; Berendsen and Hayward, 2000; He et al., 2011). As already noticed, a reliable hybrid QM/MM potential is mandatory for an accurate modeling of critical regions of protein systems, such as the GFP HBDI moiety. On the other hand, this imposes practical restraints about the simulation time which, in turn, limits the sampling of the low-frequency modes. In this section, starting from MD simulations of GFP A, I, and B-forms, a cross-correlation-based approach in the time-domain has been employed to give hints about the existence of concerted motions involving distant weak interactions in the GFP HBDI pocket. In particular, the normalized cross-correlation function *r*_*xy*_(τ) was evaluated between each H-bond in the pocket and the cavity volume and between each pair of H-bonds located on opposite sides of the HBDI moiety (one side including His148, Asn146, Thr203, and the other one including Arg96, Gln94, see Figure 2).

As observed in the previous section, the volume of the chromophore cavity is an indirect measure of the packing of the proton-wire around HBDI. To extract potential structural additional determinants affecting the volume and their equilibrium fluctuations and correlations, the most relevant H-bonds/volume cross-correlations were computed and reported in Table 1 and Supplementary Table 1. In this analysis we report significant values of the local maximum (or minimum) and the delay times at which these features appear for several cross-correlation functions between structural determinants. For example, looking first at the proton-wire H-bonds, the correlation between O(Tyr66)—O(Wat) and the volume shows a maximum value of 0.53 at 5 fs as delay time for the I form. This correlation further strengthens presenting a value of 0.62 for the B form and this time the negative −65 fs at which the maximum appears means that the volume lags behind O(Tyr66)—O(Wat) distance. The negative charge acquired by O(Tyr66) in the HBDI deprotonated forms forces the water molecule to oscillate more toward the chromophore, as a consequence of a stronger polar interaction. This reflects into a strong, direct (<100 fs delay) positive correlation for the I and the B-GFP. This result suggests that the volume is very sensitive to the change of the chromophore protonation state and its consequent change in hydrogen bond interaction with water, resulting in an important correlation between them. The short time delay found is also indicative of almost direct correlation between the two quantities. On the other hand, the O(Ser205)—O(Wat) distance appears largely uncorrelated with the volume (Supplementary Table 1). Then, the O(Ser205)—O(Glu222) H-bond shows a lag-free correlation with the pocket volume in the A-form, suggesting a fluctuation of Ser205, which is lost in the I-form. Instead, an intense anti-correlation (negative value) appears in the B-form. The >500 fs delay suggests that this negative correlation is not a direct effect, but it could reflect some delocalized motion involving larger portions of the structure. It is possible that the Glu222 is affected and involved in other motions in the B form (its spatial conformation is actually very different from both the A and I forms, see previous section), and this fact can causes a delay in the correlation of O(Ser205)—O(Glu222) with the volume. However, the intense anti-correlation suggests that the dynamics of the two residues is still involved in influencing the volume changes.

**Table 1:** Most relevant normalized cross-correlation functions between H-bonding interactions involving residues near the HBDI pocket and the pocket volume for GFP A, I, and B-forms.

	**H-bond/volume**		**H-bond/volume**
**O(Tyr66)—O(Wat)**		**O(Imid)—N(Arg96)**	
I:	0.53 (5)	I:	−0.49 (1184)
B:	0.62 (−65)		
**O(Thr203)—O(wat)**		**O(Imid)—N(Gln94)**	
I:	0.51 (1057)	A:	0.49 (−907)
B:	−0.62 (−9)		
**O(Ser205)—O(Glu222)**			
A:	0.48 (−1)		
B:	−0.66 (509)		
**N(His148)—O(Wat)**			
I:	0.49 (220)		
B:	0.64 (62)		
**N(His148)—O(Tyr66)**			
I:	0.55 (209)		
B:	0.68 (−214)		
**O(Asn146)—O(Tyr66)**			
I:	0.46 (−452) −0.48 (908)		
B:	−0.65 (335)		

*Maximum (or minimum) absolute values and the delay times (τ in fs) at which these features are found for the cross-correlations (r_xy_) are reported*.

Looking instead at the correlations between the pocket volume and the H-bonds external to the proton-wire, the O(Thr203)—O(Wat) interaction is investigated. In this case, significant correlations are found just for the anionic forms. This result supports the hypothesis that the chromophore (and its environment) interacts with Thr203 after the proton transfer reaction (i.e., once the chromophore becomes anionic). More in detail, a high negative correlation is found in the B-form. The negligible delay suggests that the intermittent interaction between the Thr203 OH group and the network water molecule (found only in the B-form) is able to move the water molecule away from the HBDI moiety. Moreover, this result suggests that the interaction with Thr203 (both in terms of degree of correlation and almost absent time-delay) is very important in the B form and that has a direct influence in modifying the volume of the chromophore pocket. On the other side, a large delay and smaller correlation is found for the I form. This fact suggests that this hydrogen bond has an impact on the volume, however it is also influenced by other collective motions and interactions causing the larger observed delay in the correlation. These trends support the hypothesis that the interactions with Thr203 is typical of the anionic forms and it has more impact in the B form that represents the “relaxed” and optimized chromophore pocket after the proton transfer reaction.

N(His148)—O(Wat) always reveals some positive correlation with the volume. Because of the geometry of the pocket, the departure of the water residue from His148 implies also an expansion of GFP main H-bond network. In analogy with the previous case, also in this case a shorter delay is found in the B form with respect to the I one suggesting again that a more direct connection is found when the environment is already rearranged for the anionic chromophore form. Less obvious is the correlation shown by N(His148)—O(Tyr66) in the I and B-forms. The ~±200 fs delays suggest some mechanism also involving residues located outside the pocket. When the correlation involves the chromophore itself, in both the I and B forms similar delays are found. In this case it is not the environment but the chromophore itself involved (anionic in both cases) and affected in the same way by motions involving other residues. The behavior is even more intricate for the O(Asn146)—O(Tyr66)/volume pair, whose correlation starts as positive in the A-form, becoming predominantly negative in the B one. Moreover, the clearly different delays for the positively correlated contribution comparing the A and I with the B-form reveal an abrupt change of the underlying collective motion. H-bonds on the HBDI imidazolinone side show on average weaker correlations with the cavity volume. Notable exceptions [I-form O(Imid)—N(Arg96), A-form O(Imid)—N(Gln94)] are characterized by high (~1 ps) delays and are likely the effect of some low-frequency collective modes. The fact that less significant cross-correlations are found when analyzing parameters from the imidazolinone side support the idea that the perturbation event, i.e., the proton transfer, takes place far in the space from it and so a lower impact on molecular rearrangement affecting the volume on this side is found.

**Figure 6:**
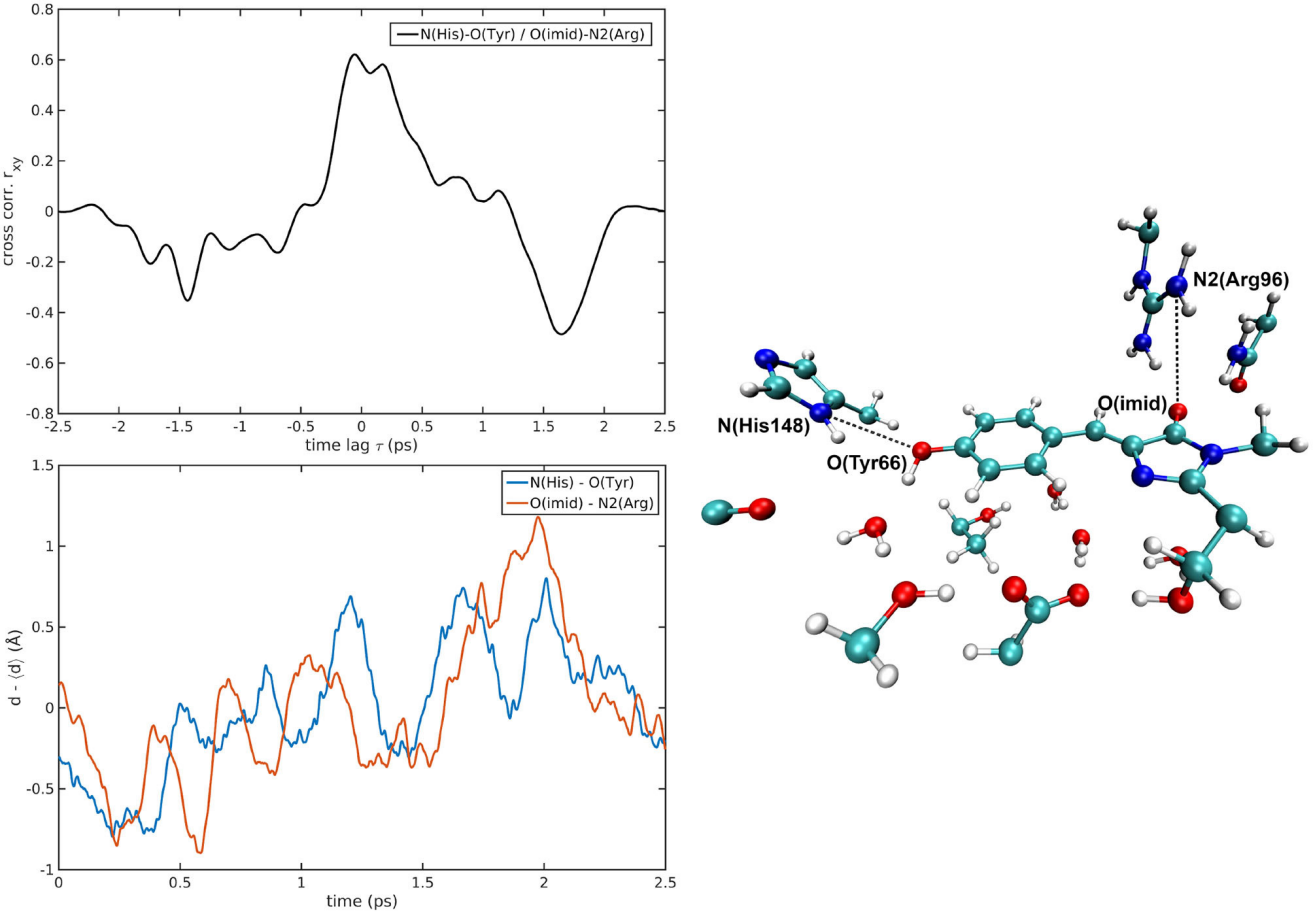
N(His148)—O(Tyr66)/O(Imid)—N2(Arg96) correlated pair in A-GFP MD [*r*_*xy*max_ = 0.62 (τ = 57 fs)].

**Figure 7:**
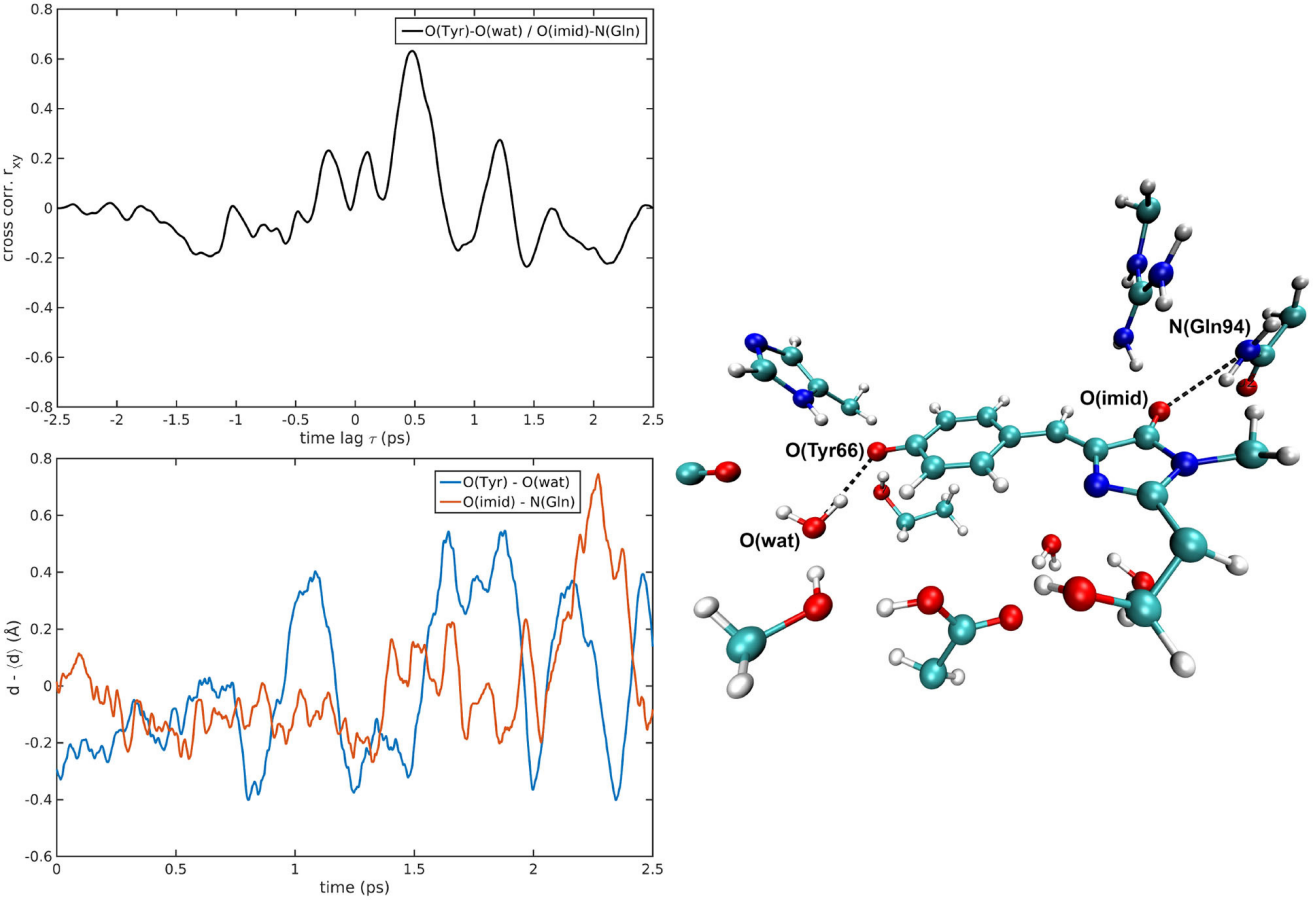
O(Tyr66)—O(wat)/O(Imid)—N(Gln94) correlated pair in I-GFP MD [*r*_*xy*max_ = 0.63 (τ = −479 fs)].

**Figure 8:**
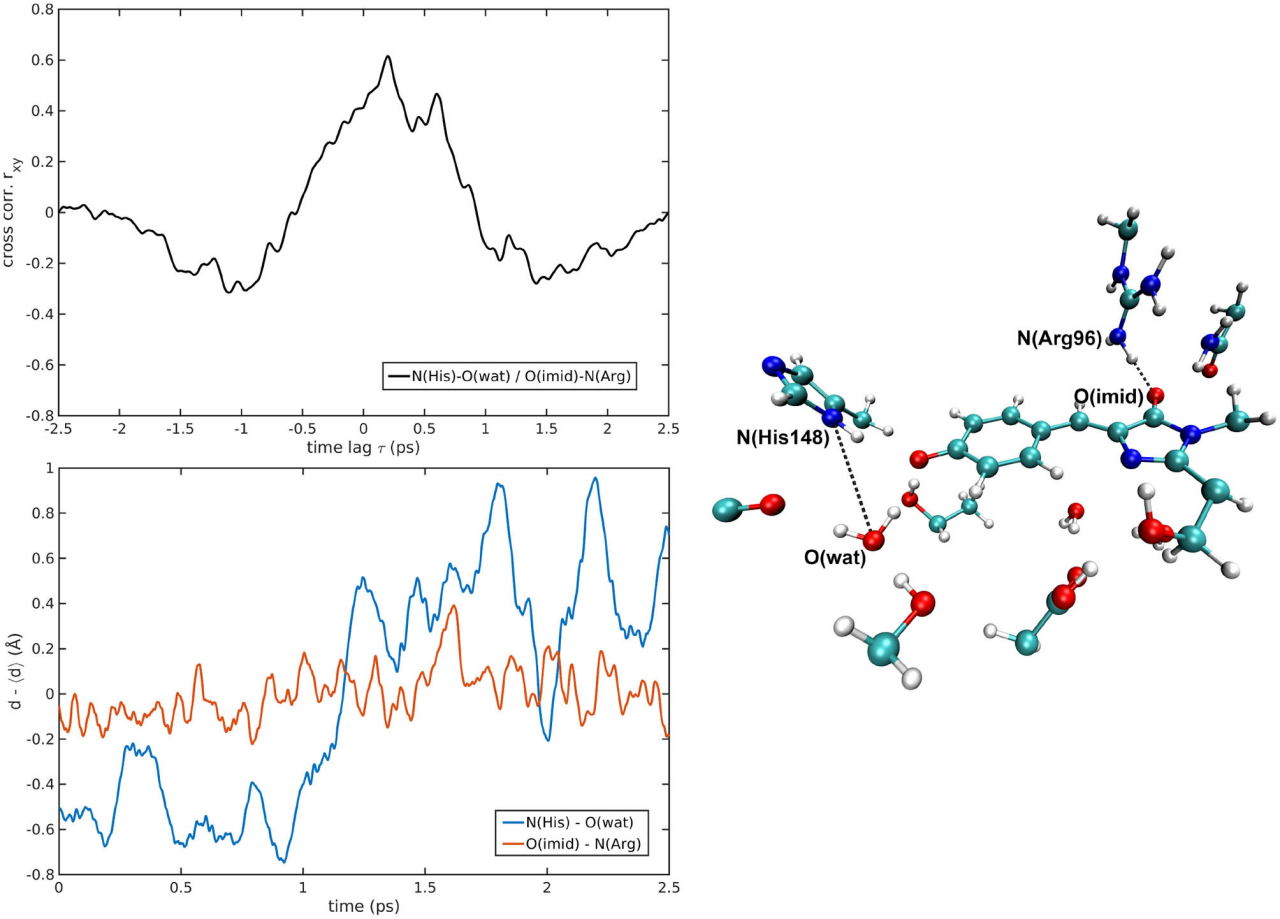
N(His148)—O(wat)/O(Imid)—N(Arg96) correlated pair in B-GFP MD [*r*_*xy*max_ = 0.61 (τ = 200 fs)].

The cross-correlations between non-covalent bond distances on distinct sides of HBDI moiety were then analyzed for regular patterns in Table 2 and Supplementary Table 2. Concerning first the main H-bond network, the O(Tyr66)—O(Wat) distance appears to be strongly related to the O(Imid)—N(Gln94) interaction in the I-form (Figure 7). O(Ser205)—O(Wat) equilibrium dynamics is quite uncorrelated with its counterparts on the opposite side. Just a similarity with O(imid)— N(Arg96) distance is found in the B-form. Interestingly, the only correlations shown by O(Ser205)—O(Glu222) [with O(Imid)—N and N2(Arg96)] are in the B-form, where the H-bond between Ser205 and Glu222 is lost (Supplementary Table 2). Distances involving residues external to the main proton-wire give rise to more evident correlations with the Imid–Arg96 or Gln94 interactions. O(Thr203)—O(Wat) exhibits strong anti-correlations with O(Imid)—N(Arg96) in both I and B-forms and with O(Imid)—N2(Arg96) and O(Imid)—N(Gln94) only in the I-form. These differences between the I and B forms could indicate that structural dynamics in different space regions of the chromophore environment are still correlated. However, this fact is more enhanced in the I form that is the intermediate situation between the stable neutral (A) and anionic (B) forms, indicating that the whole protein region has not completely adapted to new chromophore situation. N(His148)—O(Wat)/O(Imid)—N(Arg96) correlation is stronger in the I and B-forms (Figure 8). The correlation with O(Imid)—N2(Arg96) is in contrast more pronounced in the A-form. Strong correlations are kept with O(imid)—N(Gln94) in the A and I-forms, but they are lost in the B one. Looking at the N(His148)—O(Tyr66) distance, the intense correlations shown in the A (Figure 6) and I-forms with all the three interactions on the HBDI opposite side are not maintained in the anionic B form. The important role of His148 was already observed in Donati et al. (2018b), where this hydrogen bond interactions seems to be highly correlated to proton transfer event starting from the neutral form. It is possible that this interaction is still retained in the I form, but it is weakened in the B form (since it is completely relaxed after the PT), resulting in the observed absence of this correlation.

**Table 2:** Most relevant *r*_*xy*_ normalized cross-correlation functions for selected pairs of hydrogen-bonds located on opposite sides of HBDI pocket (one side including His148, Asn146, Thr203, and the other one including Arg96, Gln94, see Figure 2).

	**O(Imid)—N(Arg96)**	**O(Imid)—N2(Arg96)**	**O(Imid)—N(Gln94)**
**O(Tyr66)—O(Wat)**			
I:			0.63 (−479)
**O(Thr203)—O(Wat)**			
A:			0.48 (−14)
I:	−0.59 (−94)	0.46 (−594) −0.51 (−86)	0.48 (−70) −0.56 (−440)
B:	−0.60 (248)		
**N(His148)—O(Wat)**			
A:		0.56 (59) −0.56 (−1,675)	0.62 (26)
I:	0.60 (437) −0.46 (−1,099)		0.58 (147)
B:	0.61 (200)		
**N(His148)—O(Tyr66)**			
A:	0.49 (−463)	0.62 (57) −0.49 (−1,646)	0.65 (113)
I:	0.47 (−6) −0.55 (−1,094)	−0.49 (−1,058)	0.50 (−925)
**O(Asn146)—O(Wat)**			
B:	0.47 (213)		
A:			0.57 (495) −0.62 (−1,384)
I:	0.60 (−260)	0.52 (−94) −0.51 (−1,071)	
B:		0.46 (−651)	
**O(Asn146)—N(His148)**			
A:			0.61 (502)

*Results from A, I, and B-GFP MD simulations are shown. Maximum (or minimum) absolute values and the delay times (τ in fs) at which these features are found for the cross-correlations (r_xy_) are reported*.

O(Asn146)—O(Wat) never shows *r*_*xy*_ values above 0.5. Remarkable correlations are found with O(Imid)—N(Arg96) in the B- and with O(Imid)—N(Gln94) in the A-form. Analogously, O(Asn146)—N(His148) distance appears not so well-correlated, particularly for the B and I-forms while a high, delayed, similarity with O(imid)—N(Gln94) is nonetheless found in A-GFP. These results indicate that when dealing with correlations in a wider spatial range not involving the chromophore itself, different pairs of residues are correlated in the anionic form B and the neutral form A. Indeed, while a correlation exists between O(Asn146)—O(wat) and O(Imid)—N(Arg96) for the B form, different couples are correlated in the A form [O(Asn146)—N(His148) with O(Imid)—N(Gln94)].

In a opposite way, when the chromophore is involved all the three forms present correlations. O(Asn146)—O(Tyr66) reveals instead many highly correlated trends. While in the A-GFP the correlation with O(imid)—N(Gln94) is predominant (two equally relevant positive and negative ones with very different delays), in the I-form higher values of *r*_*xy*_ are obtained with O(imid)—N(Arg96) and O(Imid)—N2(Arg96) (in the latter case, a positive and negative correlation with distinct delays are found). Distance pairs most strongly correlated (|*r*_*xy*_| > 0.5) in each GFP state are shown in Supplementary Figures 7–10. It is interesting to underline that there is a specificity in the couples involved in the correlation with O(Asn146)—O(Tyr66), indeed while in the A form it takes place with O(imid)—N(Gln94), it switches to O(imid)—N(Arg96) and O(Imid)—N2(Arg96) for the anionic forms. So through this analysis not only correlations among residues far in the space are found, but also the specificity among the different GFP forms is disentangled.

To summarize, our results deriving from the study of the cross-correlation of key structural parameters highlight an entangled dynamics of H-bond network in the pocket and the cavity volume directly involving the chromophore, such as O(Tyr66)—O(Wat), or not, such as O(Thr203)—O(Wat). This is very interesting since it underlines the cooperativity and collective nature of structural events affecting the volume of the cavity. Moreover, the correlations found among some of the H-bonded residues located on opposite sides of the HBDI moiety [O(Tyr66)—O(Wat) with O(Imid)—N(Gln94) or N(His148)—O(Wat)/O(Imid)—N(Arg96)] reveal that the dynamics of apparently uncorrelated residues is instead characterized by an important connection, also in this case, involving or not the chromophore Tyr66 residue. These results importantly underline that disentangling the dynamics of a residue like a chromophore from its environment is tricky since it cannot be confined to it or just few residues apparently actively involved in the reaction event, but it is of crucial importance to take into account a larger portion of the protein surrounding it.

## 4. Conclusions

This paper presents the complex hydrogen bond equilibrium dynamics involving the GFP chromophore pocket in the A, I, and B forms, relying on accurate hybrid QM/MM approach for simulating the entire protein. The QM/MM ONIOM approach offers the possibility of studying in detail the effect of the protein environment on the chromophore structural and spectroscopic properties as fluorescence. A detailed molecular insight on structural differences in the active site of GFP and correlations among single hydrogen bond interactions and a macroscopic quantity, such as the volume of the chromophore cavity, is given here for the first time. In particular, the intermolecular distances involving the chromophore, such as O(Tyr66)–O(Wat) and O(Asn146)–O(Tyr66) are importantly correlated to the cavity volume in both the anionic forms. Not only the hydrogen bond network involving the chromophore, but also other side residues surrounding it are active parts in tuning the fine balance of the hydrogen bond interactions. Indeed, important differences in non-covalent interactions are found in the A, I and B forms chromophore pocket. Moreover, we found that their dynamics can correlate also residues at opposite sides of the pocket showing either a direct correlation [i.e., N(His148)–O(Tyr66) with O(Imid)–N(Arg96)] or through collective delayed and still (anti)-correlated motions [i.e., O(Thr203)–O(Wat) with O(Imid)–N(Arg96)]. Hydrogen bond pairs not close in the space also show a correlated dynamics, showing the complexity of this system where the synchrony of molecular motions has echoes in the space and that the role of apparently spectator residues is very significant instead. We directly correlate these specific interactions with the cavity volume showing a direct connection among these ones and how the volume reflects changes among the GFP forms. It is also interesting that also between the anionic species, different behaviors are found. Importantly, we were able to disentangle the coupled chromophore/neighboring residues motions over the time, and finely analyze the correlation between the entire protein pocket volume and local atomic displacements, providing a direct link among them.

## Data Availability Statement

All datasets generated for this study are included in the article/Supplementary Material.

## Author Contributions

GD, AP, and NR: project. GD: data collections. FC and FP: data analysis. All authors interpretation of data and writing.

## Conflict of Interest

FC, FP, NR, and GD declare that received financial support from Gaussian Inc. The funder was not involved in the study design, collection, analysis, interpretation of data, the writing of this article or the decision to submit it for publication. The remaining author declares that the research was conducted in the absence of any commercial or financial relationships that could be construed as a potential conflict of interest.
