# Supplementary material for: A Not Obvious Correlation Between the Structure of Green Fluorescent Protein Chromophore Pocket and Hydrogen Bond Dynamics: A Choreography From ab initio Molecular Dynamics
Source: Front Mol Biosci. 2020 Oct 27;7:569990. doi: 10.3389/fmolb.2020.569990 (PMC7653547; doi:10.3389/fmolb.2020.569990)
Supplement: Supplementary file 1 [file Data_Sheet_1.PDF]

Supplementary Material

1 SUPPLEMENTARY DATA

1.1 Figures

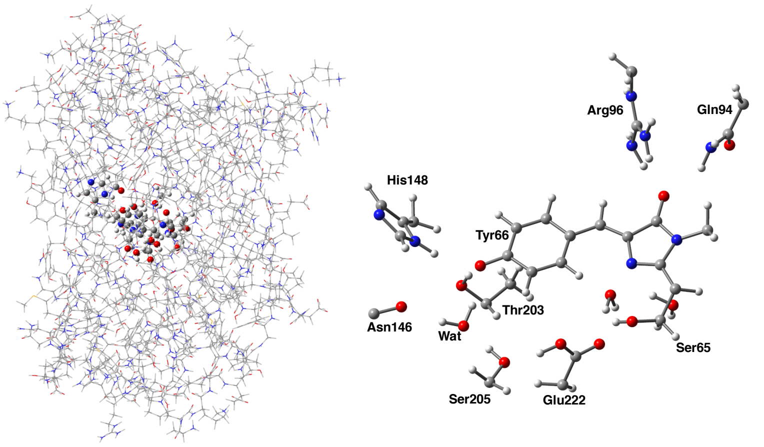

Figure S1. Left: Hybrid ONIOM (QM/MM) partition scheme of the anionic (I-form) GFP structure (PDB code: 1EMA). Right: ONIOM high level model (ball and stick representation) which includes the GFP chromophore, side chains of aminoacids involved in the hydrogen bond network - crystallographic water molecule, Ser205, Glu222 and Ser65 - and polar fragments of residues that mimic environmental effects.

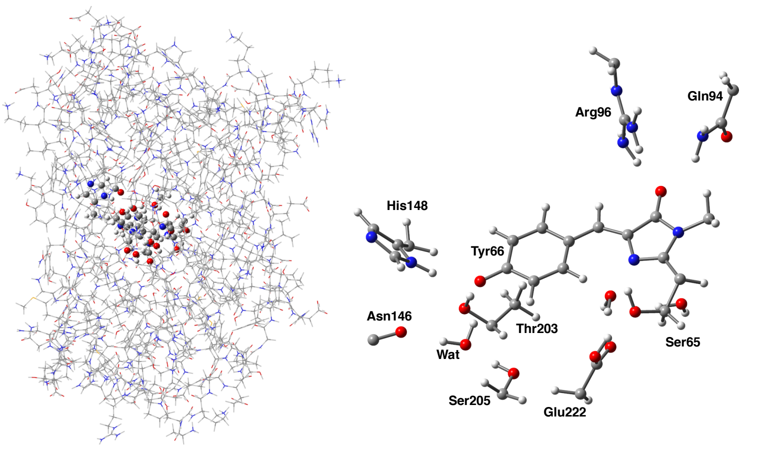

Figure S2. Left: Hybrid ONIOM (QM/MM) partition scheme of the anionic (B-form) GFP structure (PDB code: 1EMA). Right: ONIOM high level model (ball and stick representation) which includes the GFP chromophore, side chains of aminoacids involved in the hydrogen bond network - crystallographic water molecule, Ser205, Glu222 and Ser65 - and polar fragments of residues that mimic environmental effects.

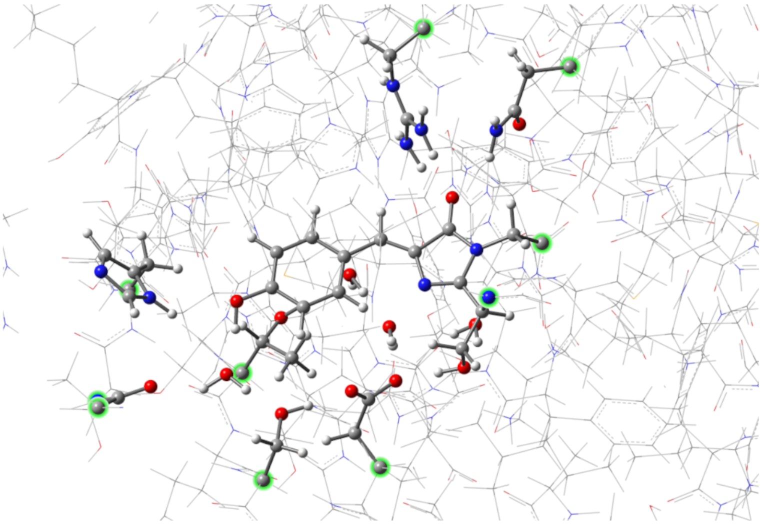

Figure S3. Detail of the QM (ball and stick representation, *Model* system) partition inside the wt-GFP protein (fine line representation, *Real* system). Link-atoms as Hydrogen species have been added to the atoms highlighted in green while retaining the original coordinates.

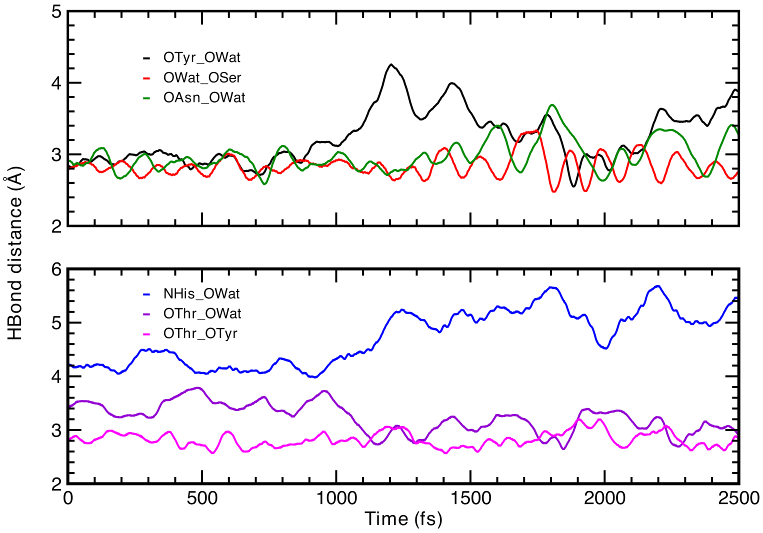

Figure S4. Time series of the hydrogen bond pair distances around the Tyr66 residue and the nearby water molecule for the anionic B-form. Please see Fig.S2 for deatils. The inset shows the labels and the color scheme used.

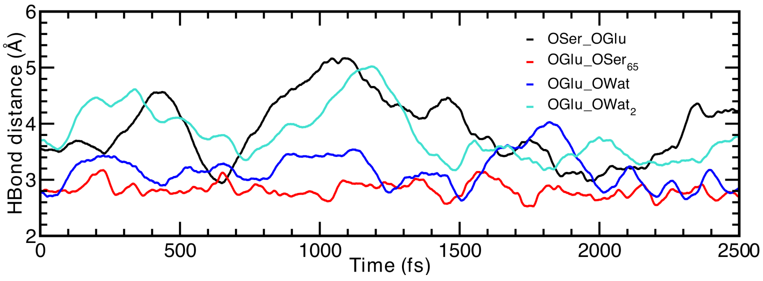

Figure S5. Time series of the hydrogen bond pair distances around the Glu222 residue for the anionic B-form. Please see Fig.S2 for deatils. The inset shows the labels and the color scheme used.

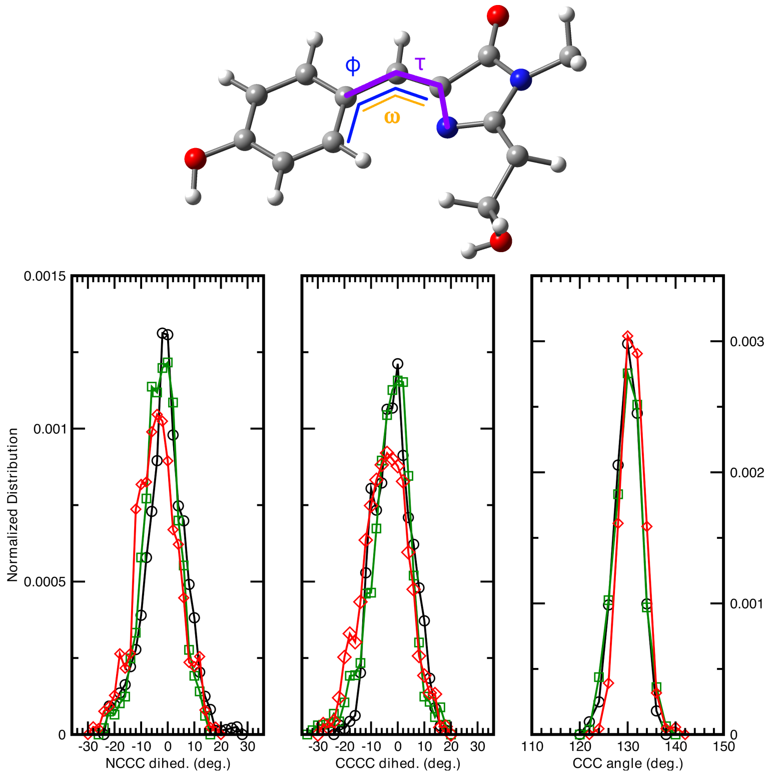

Figure S6. NCCC(τ), CCCC(ϕ) dihedral angles and CCC(ω) bond angle distributions computed for the neutral (A, black line and circles) and the anionic forms (I, green line and squares; B, red and diamonds) at 2.00° resolution. NCCC(τ): -2°; 0°, -4° for A, I and B forms. CCCC(ϕ): 0° for all cases. In the CCC case the sharp distribution is peaked at 128° for the three forms. The composition of the structural parameters is shown in color above the graphs.

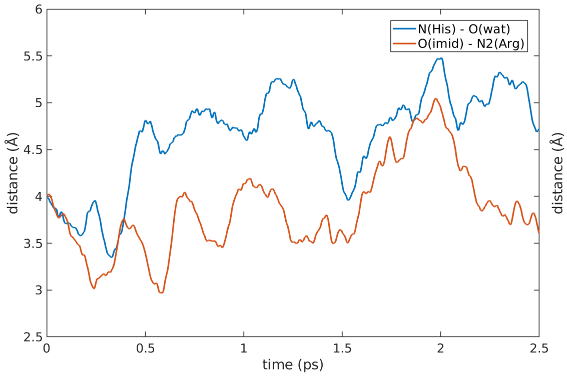

$$r_{xy\,\mathrm{max}} = 0.56\,(\tau = 59\,\mathrm{fs})$$
$$r_{xy\,\mathrm{min}} = -0.56\,(\tau = -1675\,\mathrm{fs})$$

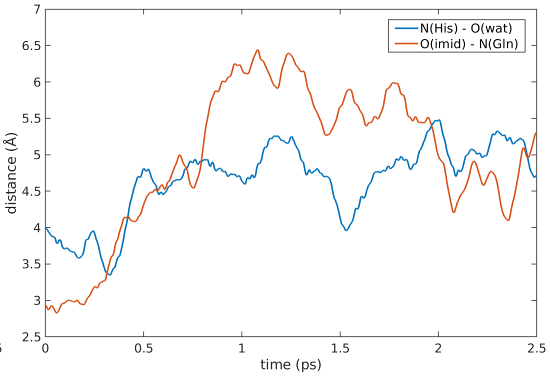

$$r_{xy\,\mathrm{max}} = 0.62\,(\tau = 26\,\mathrm{fs})$$
$$r_{xy\,\mathrm{min}} = -0.41\,(\tau = -929\,\mathrm{fs})$$

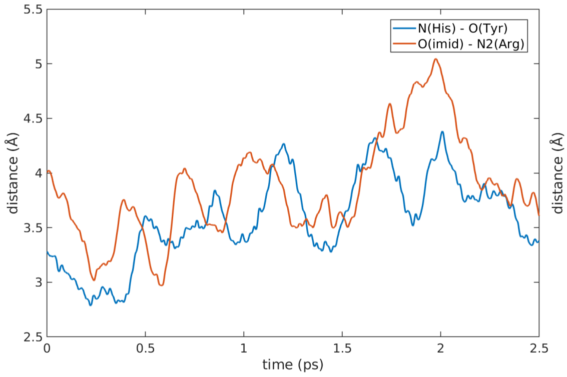

$$r_{xy\,\mathrm{max}} = 0.62\,(\tau = 57\,\mathrm{fs})$$
$$r_{xy\,\mathrm{min}} = -0.49\,(\tau = -1646\,\mathrm{fs})$$

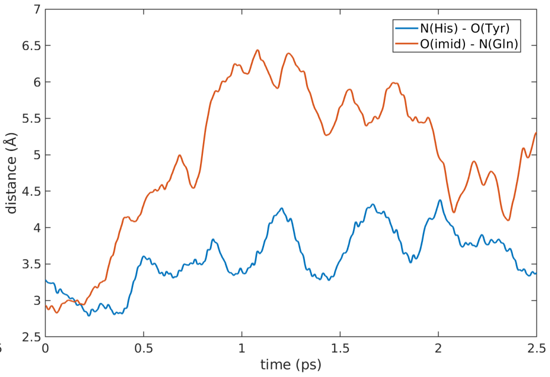

$$r_{xy\,\mathrm{max}} = 0.65\,(\tau = 113\,\mathrm{fs})$$
$$r_{xy\,\mathrm{min}} = -0.39\,(\tau = 872\,\mathrm{fs})$$

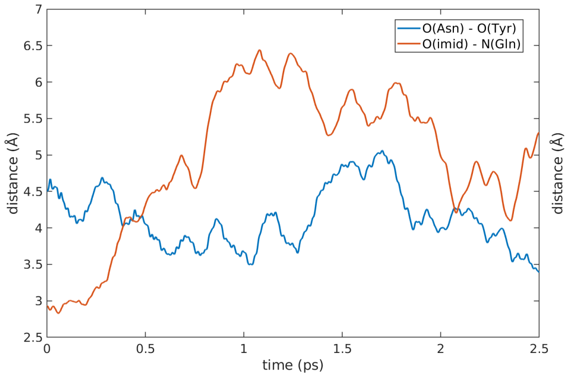

$$r_{xy\,\mathrm{max}} = 0.57\,(\tau = 495\,\mathrm{fs})$$
$$r_{xy\,\mathrm{min}} = -0.62\,(\tau = -1384\,\mathrm{fs})$$

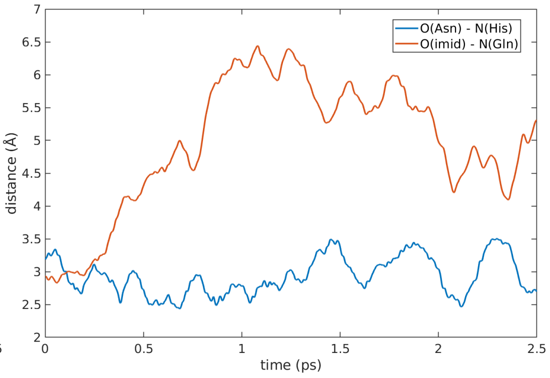

$$r_{xy\,\mathrm{max}} = 0.61\,(\tau = 502\,\mathrm{fs})$$
$$|r_{xy\,\mathrm{min}}| < 0.35$$

Figure S7. Correlated atom distances belonging to opposite side of GFP HBDI cavity in GFP A-form.

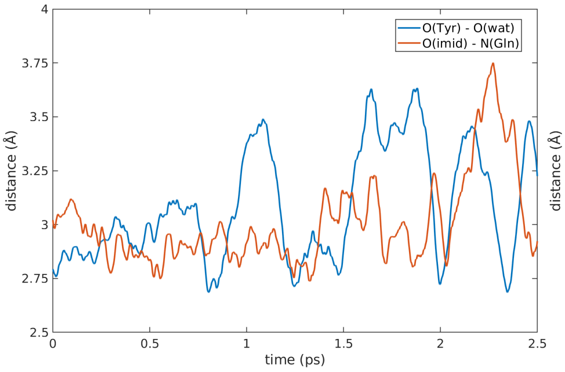

$$r_{xy\,\mathrm{max}} = 0.63\,(\tau = -479\,\mathrm{fs})$$
$$|r_{xy\,\mathrm{min}}| < 0.35$$

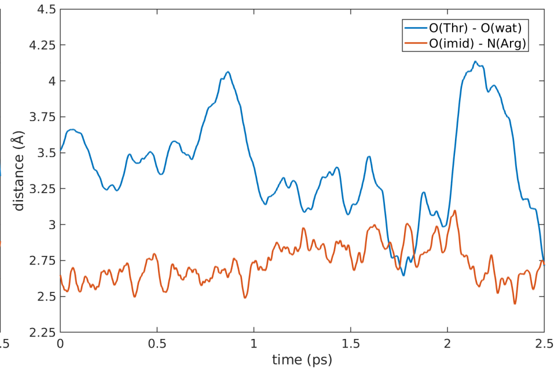

$$r_{xy\,\mathrm{max}} < 0.35$$
$$r_{xy\,\mathrm{min}} = -0.59\,(\tau = -94\,\mathrm{fs})$$

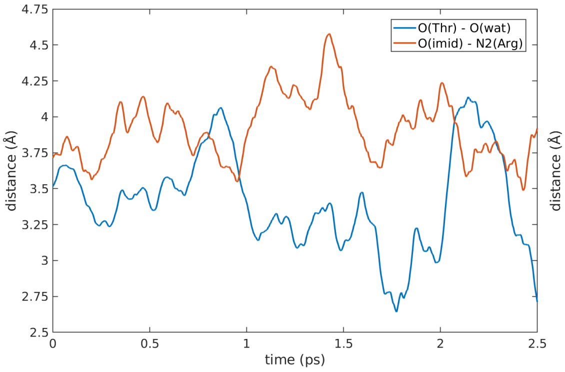

$$r_{xy\,\mathrm{max}} = 0.46\,(\tau = -594\,\mathrm{fs})$$
$$r_{xy\,\mathrm{min}} = -0.51\,(\tau = -86\,\mathrm{fs})$$

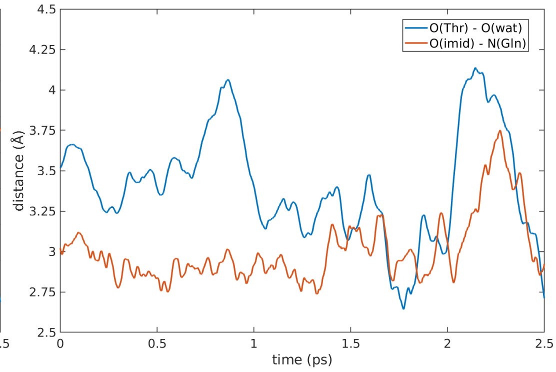

$$r_{xy\,\mathrm{max}} = 0.48\,(\tau = -70\,\mathrm{fs})$$
$$r_{xy\,\mathrm{min}} = -0.56\,(\tau = -440\,\mathrm{fs})$$

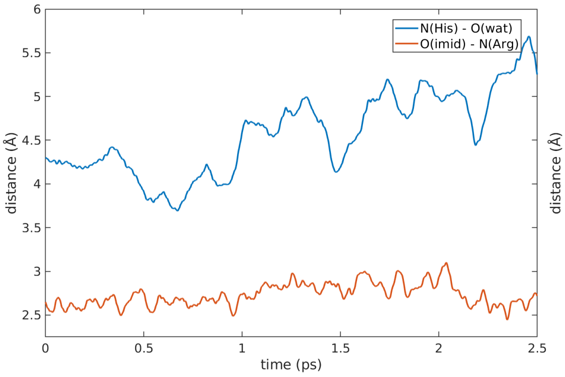

$$r_{xy\,\mathrm{max}} = 0.60\,(\tau = 437\,\mathrm{fs})$$
$$r_{xy\,\mathrm{min}} = -0.46\,(\tau = -1099\,\mathrm{fs})$$

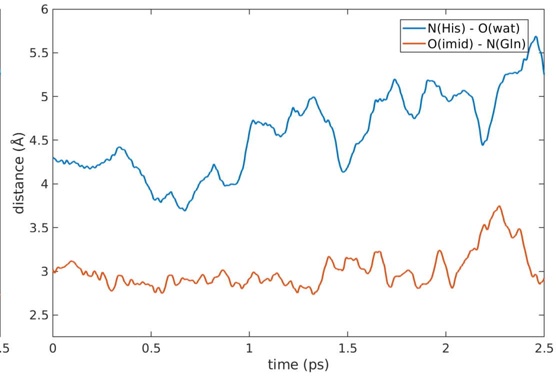

$$r_{xy\,\mathrm{max}} = 0.58\,(\tau = 147\,\mathrm{fs})$$
$$r_{xy\,\mathrm{min}} = -0.39\,(\tau = -1629\,\mathrm{fs})$$

Figure S8. Correlated atom distances belonging to opposite side of GFP HBDI cavity in GFP I-form.

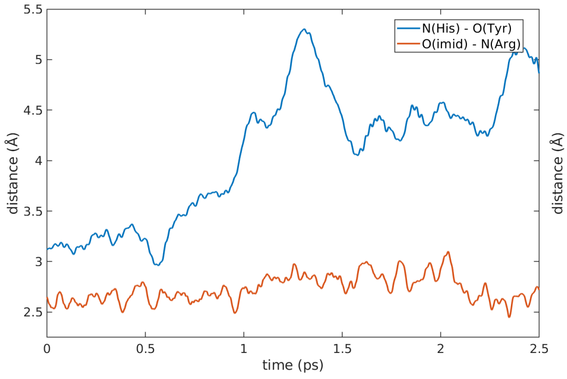

$$r_{xy\,\max} = 0.47\,(\tau = -6\,\text{fs})$$
$$r_{xy\,\min} = -0.55\,(\tau = -1094\,\text{fs})$$

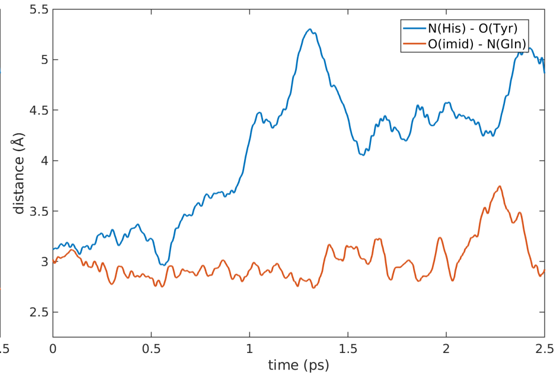

$$r_{xy\,\max} = 0.50\,(\tau = -925\,\text{fs})$$
$$r_{xy\,\min} = -0.36\,(\tau = -2075\,\text{fs})$$

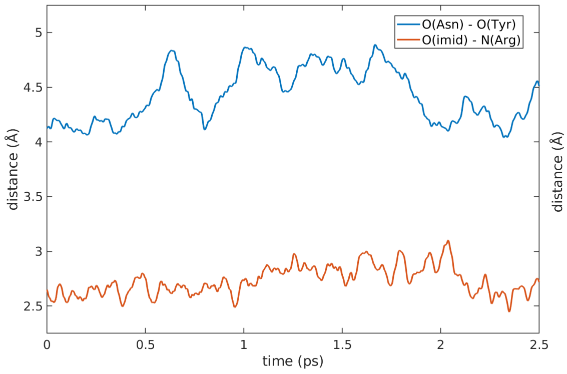

$$r_{xy\,\max} = 0.60\,(\tau = -260\,\text{fs})$$
$$r_{xy\,\min} = -0.39\,(\tau = 738\,\text{fs})$$

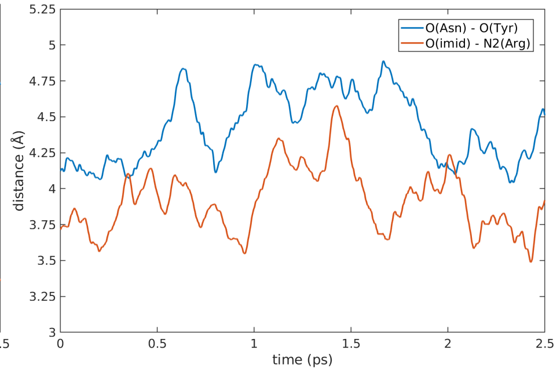

$$r_{xy\,\max} = 0.52\,(\tau = -94\,\text{fs})$$
$$r_{xy\,\min} = -0.51\,(\tau = -1071\,\text{fs})$$

Figure S9. Correlated atom distances belonging to opposite side of GFP HBDI cavity in GFP I-form (cont.).

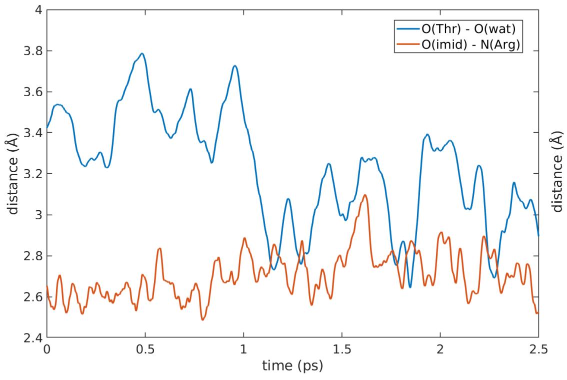

$$r_{xy\,\max} < 0.35$$
$$r_{xy\,\min} = -0.60\,(\tau = 248\,\text{fs})$$

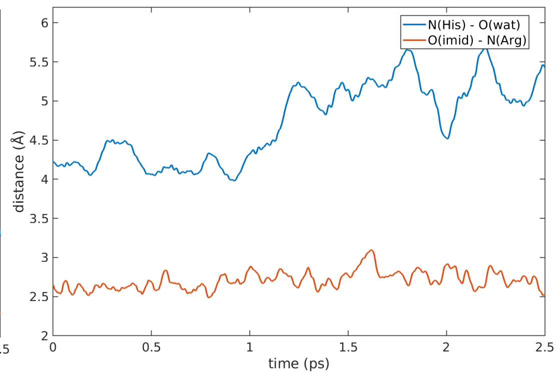

$$r_{xy\,\max} = 0.61\,(\tau = 200\,\text{fs})$$
$$|r_{xy\,\min}| < 0.35$$

Figure S10. Correlated atom distances belonging to opposite side of GFP HBDI cavity in GFP B-form.

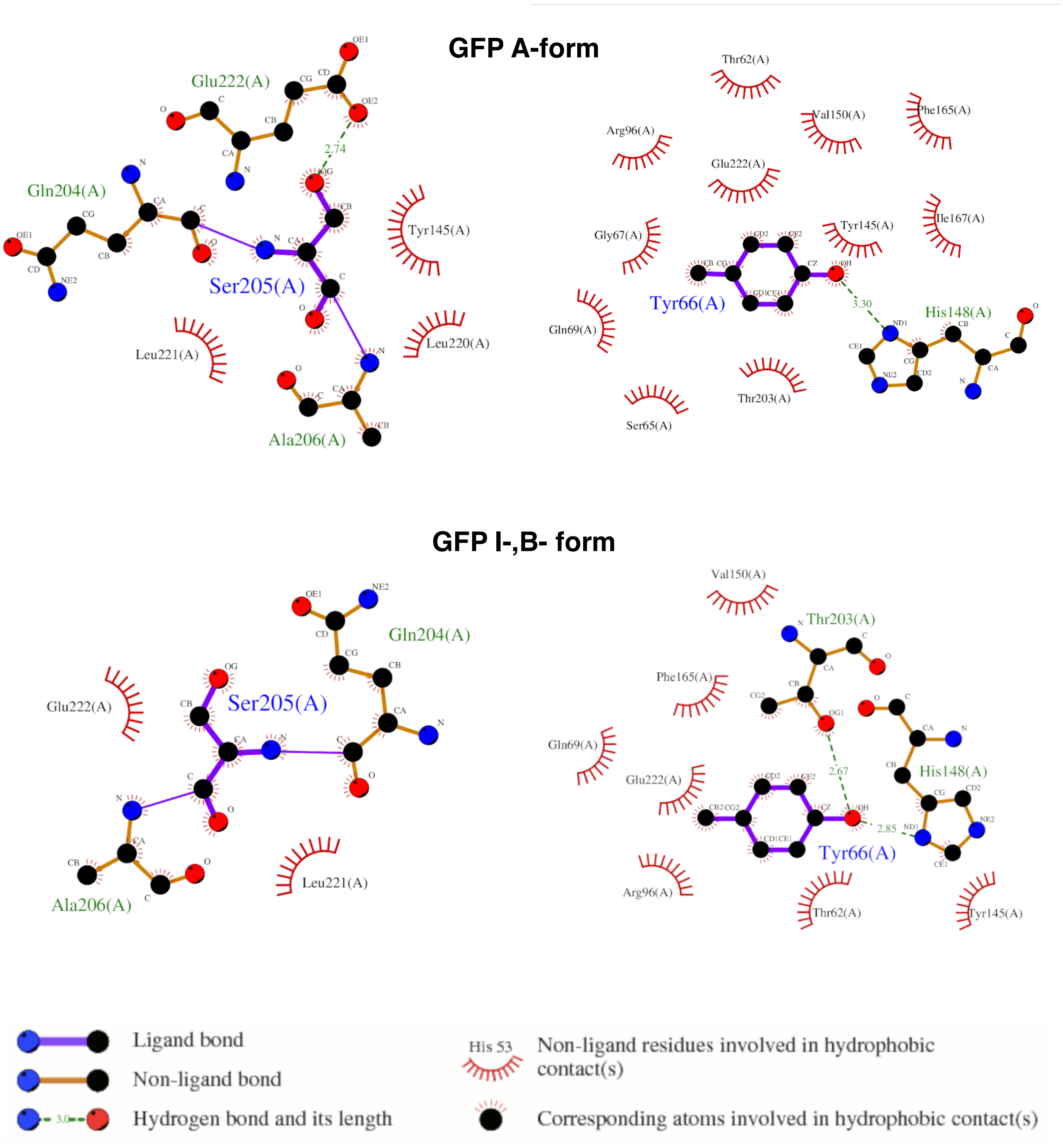

Figure S11. Interaction maps (generated by LigplotWallace et al. (1995)) of both neutral GFP A form using the crystallographic structure of wild type GFP (wt-GFP) form *Aequorea Victoria* jellyfish (PDB code: 1GFL, upper panel) (Yang et al., 1996) and of the GFP mutant S65T (PDB ID: 1EMA) (Ormö et al., 1996) belonging to class 2 (EGFPs), considered as a starting model for the anionic GFP forms. Thus, this one was used for modeling both the anionic species (I-, B-forms).

1.2 Tables

Table S1. Normalized cross-correlations between H-bonds of HBDI pocket and the pocket volume for GFP A,I and B-forms. Data are reported as $r_{xy\ \mathrm{max/min}}(\tau)$.

set1 H-bond/volume		set2 H-bond/volume		
O(Tyr) – O(Thr)	–	O(imid) – N(Arg)	–	A-GFP
	0.35 (53)		0.40 (-30) -0.49 (1184)	I-GFP
	–		0.36 (15)	B-GFP
O(Tyr) – O(wat)	0.35 (15)	O(imid) – N2(Arg)	–	
	0.53 (5)		0.37 (97) -0.39 (638)	
	0.62 (-65) -0.41 (744)		–	
O(Thr) – O(wat)	–	O(imid) – N(Gln)	0.49 (-907)	
	0.51 (1057)		0.43 (1103)	
	-0.62 (-9)		–	
O(Ser) – O(wat)	0.40 (8)			
	–			
	–			
O(Ser) – O(Glu)	0.48 (-1)			
	–			
	0.35 (1395) -0.66 (509)			
N(His) – O(wat)	0.40 (40)			
	0.49 (220) -0.35 (1631)			
	0.64 (62)			
N(His) – O(Tyr)	0.35 (-688)			
	0.55 (209) -0.37 (-1023)			
	0.68 (-214) -0.37 (1615)			
O(Asn) – O(wat)	0.38 (-810)			
	0.39 (644)			
	0.39 (46)			
O(Asn) – O(Tyr)	0.42 (-491) -0.38 (164)			
	0.46 (-452) -0.48 (908)			
	0.44 (1210) -0.65 (335)			
O(Asn) – N(His)	0.42 (-421)			
	–			
	–			

Table S2. r_{xy} normalized cross-correlations and the corresponding time-lags τ (fs) for selected pairs of hydrogen-bonds located on opposite sides of HBDI pocket. Results from A, I and B-GFP MD simulations are shown. Data are reported as $r_{xy\,\text{max/min}}(\tau)$.

	O(imid) – N(Arg)	O(imid) – N2(Arg)	O(imid) – N(Gln)	
O(Tyr) – O(Thr)	–	0.41 (424) -0.37 (52)	0.39 (1484)	A-GFP
	–	–	-0.40 (199)	I-GFP
	–	–	–	B-GFP
O(Tyr) – O(wat)	–	–	–	
	0.38 (490)	0.40 (434) -0.45 (-582)	0.63 (-479)	
	0.40 (-412)	-0.42 (-1139)	0.36 (-1193) -0.37 (689)	
O(Thr) – O(wat)	0.36 (-749)	0.45 (478)	0.48 (-14)	
	-0.59 (-94)	0.46 (-594) -0.51 (-86)	0.48 (-70) -0.56 (-440)	
	-0.60 (248)	0.42 (-1166)	0.41 (634) -0.38 (-1254)	
O(Ser) – O(wat)	–	–	–	
	–	–	–	
	0.41 (125)	–	–	
O(Ser) – O(Glu)	–	–	–	
	–	–	–	
	0.40 (-593) -0.35 (368)	0.44 (-261)	–	
N(His) – O(wat)	0.39 (-788) -0.37 (-1836)	0.56 (59) -0.56 (-1675)	0.62 (26) -0.41 (-929)	
	0.60 (437) -0.46 (-1099)	0.44 (542) -0.44 (-530)	0.58 (147) -0.39 (-1629)	
	0.61 (200)	–	0.35 (-632) -0.36 (612)	
N(His) – O(Tyr)	0.49 (-463)	0.62 (57) -0.49 (-1646)	0.65 (113) -0.39 (872)	
	0.47 (-6) -0.55 (-1094)	-0.49 (-1058)	0.50 (-925) -0.36 (-2075)	
	0.38 (-169) -0.39 (-1466)	-0.37 (-1030)	–	
O(Asn) – O(wat)	0.39 (-173)	0.41 (-273) -0.36 (-1761)	0.45 (143)	
	0.35 (509)	0.40 (536)	0.40 (150)	
	0.47 (213)	–	0.38 (-583)	
O(Asn) – O(Tyr)	0.38 (-188)	0.45 (-320)	0.57 (495) -0.62 (-1384)	
	0.60 (-260) -0.39 (738)	0.52 (-94) -0.51 (-1071)	0.45 (-898) -0.35 (74)	
	0.37 (-618)	0.46 (-651)	–	
O(Asn) – N(His)	0.44 (-252)	0.41 (326)	0.61 (502)	
	-0.36 (-473)	–	–	
	–	–	0.38 (-1164) -0.36 (-462)	

REFERENCES

Ormö, M., Cubitt, A. B., Kallio, K., Gross, L. A., Tsien, R. Y., and Remington, S. J. (1996). Crystal structure of the aequorea victoria green fluorescent protein. *Science* 273, 1392–1395

Wallace, A. C., Laskowski, R. A., and Thornton, J. M. (1995). Ligplot: a program to generate schematic diagrams of protein-ligand interactions. *Protein engineering, design and selection* 8, 127–134

Yang, F., Moss, L. G., and Phillips, G. N. (1996). The molecular structure of green fluorescent protein. *Nat. Biotechnol.* 14, 1246–1251
